# Seagrass-mediated rhizosphere redox gradients are linked with ammonium accumulation driven by diazotrophs

**DOI:** 10.1128/spectrum.03335-23

**Published:** 2024-03-01

**Authors:** Kasper Elgetti Brodersen, Maria Mosshammer, Meriel J. Bittner, Søren Hallstrøm, Jakob Santner, Lasse Riemann, Michael Kühl

**Affiliations:** 1 Marine Biological Section, Department of Biology, University of Copenhagen, Helsingør, Denmark; 2 Department of Crop Sciences, Institute of Agronomy, University of Natural Resources and Life Sciences Vienna, Tulln an der Donau, Austria; Universidad Nacional Autonoma de Mexico - Campus Morelos, Cuernavaca, Mexico

**Keywords:** ammonium, diazotrophs, redox conditions, rhizosphere, seagrass

## Abstract

Seagrasses can enhance nutrient mobilization in their rhizosphere via complex interactions with sediment redox conditions and microbial populations. Yet, limited knowledge exists on how seagrass-derived rhizosphere dynamics affect nitrogen cycling. Using optode and gel-sampler-based chemical imaging, we show that radial O_2_ loss (ROL) from rhizomes and roots leads to the formation of redox gradients around below-ground tissues of seagrass (*Zostera marina*), which are co-localized with regions of high ammonium concentrations in the rhizosphere. Combining such chemical imaging with fine-scale sampling for microbial community and gene expression analyses indicated that multiple biogeochemical pathways and microbial players can lead to high ammonium concentration within the oxidized regions of the seagrass rhizosphere. Symbiotic N_2_-fixing bacteria (*Bradyrhizobium*) were particularly abundant and expressed the diazotroph functional marker gene *nifH* in *Z. marina* rhizosphere areas with high ammonium concentrations. Such an association between *Z. marina* and *Bradyrhizobium* can facilitate ammonium mobilization, the preferred nitrogen source for seagrasses, enhancing seagrass productivity within nitrogen-limited environments. ROL also caused strong gradients of sulfide at anoxic/oxic interfaces in rhizosphere areas, where we found enhanced *nifH* transcription by sulfate-reducing bacteria. Furthermore, we found a high abundance of methylotrophic and sulfide-oxidizing bacteria in rhizosphere areas, where O_2_ was released from seagrass rhizomes and roots. These bacteria could play a beneficial role for the plants in terms of their methane and sulfide oxidation, as well as their formation of growth factors and phytohormones. ROL from below-ground tissues of seagrass, thus, seems crucial for ammonium production in the rhizosphere via stimulation of multiple diazotrophic associations.

**IMPORTANCE:**

Seagrasses are important marine habitats providing several ecosystem services in coastal waters worldwide, such as enhancing marine biodiversity and mitigating climate change through efficient carbon sequestration. Notably, the fitness of seagrasses is affected by plant–microbe interactions. However, these microscale interactions are challenging to study and large knowledge gaps prevail. Our study shows that redox microgradients in the rhizosphere of seagrass select for a unique microbial community that can enhance the ammonium availability for seagrass. We provide first experimental evidence that *Rhizobia*, including the symbiotic N_2_-fixing bacteria *Bradyrhizobium*, can contribute to the bacterial ammonium production in the seagrass rhizosphere. The release of O_2_ from rhizomes and roots also caused gradients of sulfide in rhizosphere areas with enhanced nifH transcription by sulfate-reducing bacteria. O_2_ release from seagrass root systems thus seems crucial for ammonium production in the rhizosphere via stimulation of multiple diazotrophic associations.

## INTRODUCTION

Seagrasses are marine flowering plants that are important ecosystem engineers providing vital services to coastal regions worldwide, such as enhancing fisheries production (1), removing seawater pollutants (2, 3), and driving sequestration of carbon from the atmosphere (4, 5). Below-ground tissue of seagrasses releases O_2_ (termed radial O_2_ loss; ROL) (6–9) and high concentrations of dissolved organic carbon (DOC) (10–12) into the rhizosphere. This leads to chemical and biological re-oxidation of sediment-produced H_2_S within oxic microzones (13, 14) and stimulation of the rhizosphere microbial community (14–18). ROL is achieved via an extensive internal lacunar system that enables effective long-distance gas transport from leaf to rhizosphere in seagrasses (19–21). Leaf photosynthesis enhances the below-ground tissue oxidation capacity (9, 13, 22), enabling seagrasses to inhabit hostile, sulfide-rich marine environments (23–25). Inadequate internal plant aeration, mainly as a result of low water-column O_2_ availability during night-time, can lead to intrusion of phytotoxic H_2_S (20, 26–29), increasing seagrass mortality (30), which can trigger large-scale die-off events (23, 31). The seagrass rhizosphere is thus characterized by a mosaic of plant-driven chemical microgradients (13, 32–35) that, together with rhizosphere-associated microbes, facilitate detoxification of sediment-derived phytotoxins (13, 14, 35, 36), sediment acidification (14, 34, 37), and nutrient mobilization (34).

The microbial community composition in the seagrass rhizosphere is dominated by microbes involved in the sulfur cycle, such as sulfate-reducing bacteria (SRB) and sulfide-oxidizing bacteria (SOB) (35, 36, 38–41). This is a paradox, since SRB are responsible for the production of phytotoxic H_2_S in seagrass sediments through mineralization of organic matter via sulfate reduction (42–45). High rates of microbial sulfate reduction are a result of the reduced nature of seagrass sediments coupled with high levels of sulfate in seawater (46) and seagrass secretion of root and rhizome exudates (10, 11). Many SRB are capable of dinitrogen (N_2_)-fixation, whereby N_2_ is reduced to ammonium (NH_4_^+^), the preferred nitrogen source of seagrasses (16, 18). In silicate-rich coastal sediment (often observed in temperate seagrass ecosystems), nitrogen is often the limiting nutrient (47–49). Seagrass rhizosphere sediments can exhibit intense N_2_ fixation (15, 16, 18, 43, 44, 50), and heterotrophic, diazotrophic bacteria (especially N_2_-fixing SRB) have been hypothesized to thrive in symbiosis with seagrasses in the rhizosphere, based on carbon and nitrogen exchange (16). In the rhizosphere of *Zostera noltii*, root- and rhizome-associated N_2_ fixation rates were largely driven by SRB, reaching 40- to 140-fold higher rates than in the bulk sediment (BS) and accounting for ~30% of the fixed nitrogen in the rhizosphere (18). Furthermore, an endosymbiotic N_2_-fixing bacterium (Candidatus Celerinatantimonas neptuna) was recently found in the roots of the seagrass *Posidonia oceanica*, where the bacterium exchanges ammonia and amino acids for sugars with its seagrass host (51).

Large knowledge gaps about how seagrasses interact with their rhizosphere microbial community still exist, especially in relation to the accumulation of plant-available ammonium in nitrogen-limited sediment environments. In terrestrial flowering plants, such as legumes, symbiotic N_2_-fixing *Rhizobia* are essential for efficient nutrient mobilization and assimilation (52–54), but it is unknown whether similar symbiotic relationships between seagrasses and rhizosphere-associated rhizobium bacteria have evolved.

In the present study, we used a novel combination of high-resolution sampling and biogeochemical measurements to show that strong redox microgradients in the rhizosphere of the seagrass (*Z. marina* L.) select for a unique microbial community that enhances the ammonium availability for the seagrass host. Our results provide the first experimental evidence that *Rhizobia*, including the symbiotic N_2_-fixing bacteria *Bradyrhizobium*, can contribute to the bacterial ammonium production in the seagrass rhizosphere.

## RESULTS AND DISCUSSION

### Oxic microhabitats and pH microheterogeneity in the seagrass rhizosphere

**Fig 1:**
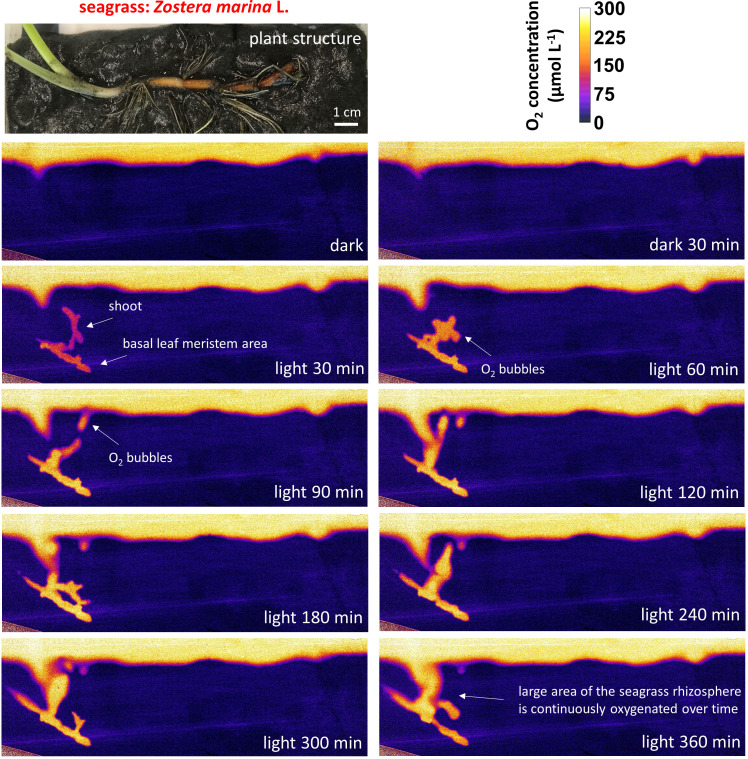
O_2_ distribution and dynamics in the seagrass rhizosphere. The *Z. marina* plant structures and position are shown in the upper panel. Color-coded images show the O_2_ concentration in the seagrass rhizosphere and sediment in response to a dark/light transition. Legend depicts the color-coded O_2_ concentration in µmol L^−1^.

**Fig 2:**
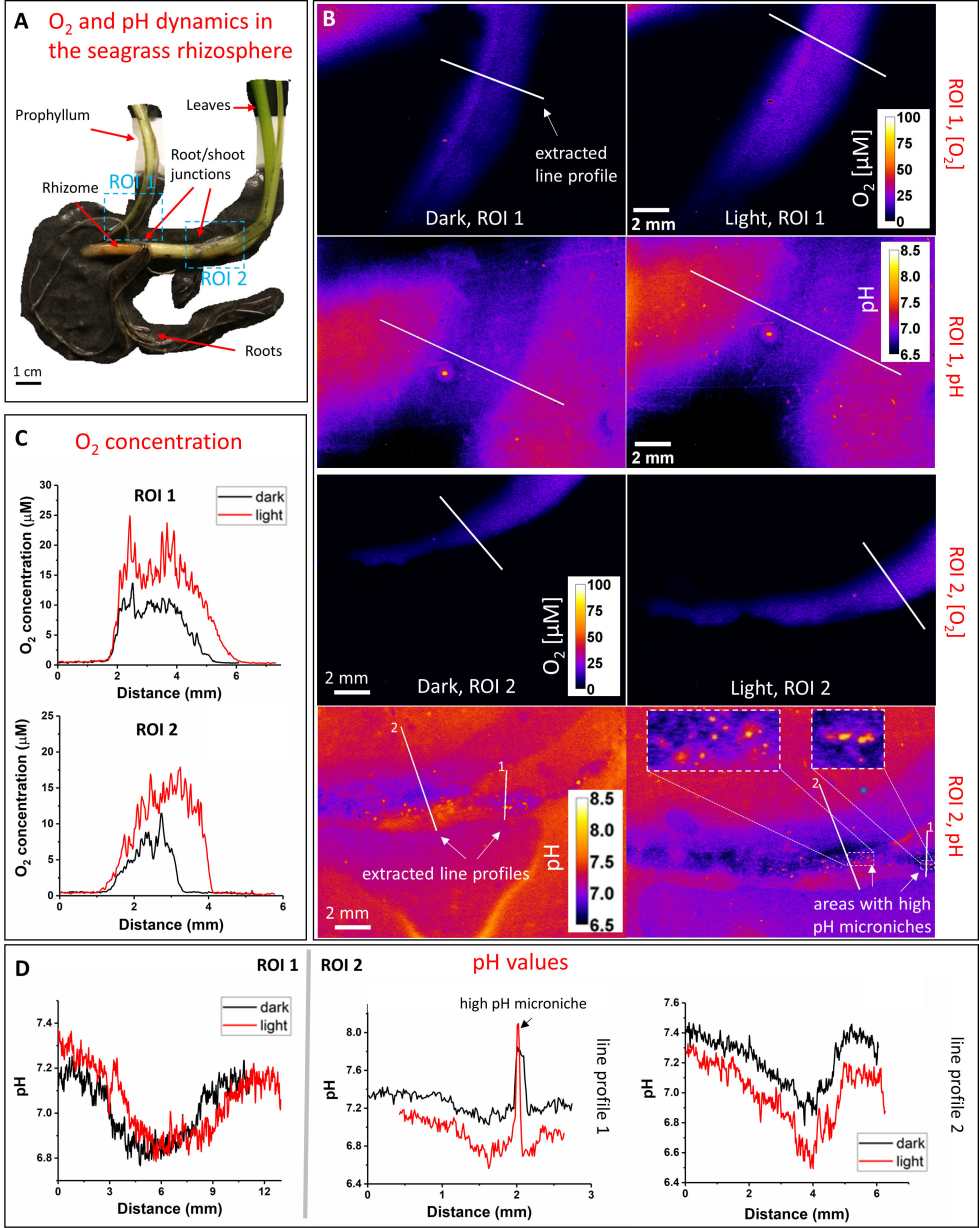
O_2_ and pH dynamics in the seagrass rhizosphere. (**A**) *Z. marina* plant structure and position of selected regions of interest (ROI 1 and 2). (**B**) Color-coded images of the O_2_ and pH distribution around the below-ground tissue within ROI 1 and 2, where the figure legend depicts the O_2_ concentration and the pH value, respectively. (**C–D**) Extracted line profiles (as shown on color-coded images) showing the cross tissue O_2_ concentration and pH values as a response to changing dark and light conditions. Biological replication can be found in Fig. S6 and S7.

In the light, ROL from the below-ground tissues of *Z. marina*, especially around the basal leaf meristem region, oxygenated particular areas of the seagrass rhizosphere (Fig. 1; Fig. S6). While such below-ground tissue oxidation capacity seemed to cease in darkness for some *Z. marina* plants (Fig. 1), other plants showed ROL from the below-ground tissue in both light and darkness (Fig. S6 and S7), albeit with less ROL in darkness. ROL was also observed around older parts of the root system, for example, the rhizome-end (Fig. 1) and older root/shoot junctions, where single leaves arising from the rhizome (i.e., prophyllums) produced O_2_ via photosynthesis in the light and functioned as a pathway for O_2_ transport in darkness (Fig. 2; Fig. S6 and S7). Our measurements are in alignment with previously reported ROL patterns for below-ground tissues of *Z. marina* seagrasses (8, 9, 13).

The ROL from the below-ground tissues of *Z. marina* led to co-localized rhizosphere acidification (Fig. 2; Fig. S6 and S7) due to sulfide re-oxidation (14, 55). However, several high pH microenvironments were also present around the rhizome, especially around the rhizome nodes and root junctions (Fig. 2; Fig. S6 and S7) indicative of proton-consuming processes such as sulfate reduction (37). Such pH microheterogeneity in the seagrass rhizosphere and the observed higher below-ground tissue acidification capacity during leaf photosynthetic O_2_ production in light are similar to previous findings (13, 34, 37). Seagrass-derived, low-pH microenvironments are vital for nutrient mobilization in carbonate-rich sediments with strong phosphorus fixation capacity (56, 57), via protolytic calcium phosphate dissolution leading to phosphorus solubilization (34).

### Redox gradients affect the distribution of sulfide and ammonium in the seagrass rhizosphere

**Fig 3:**
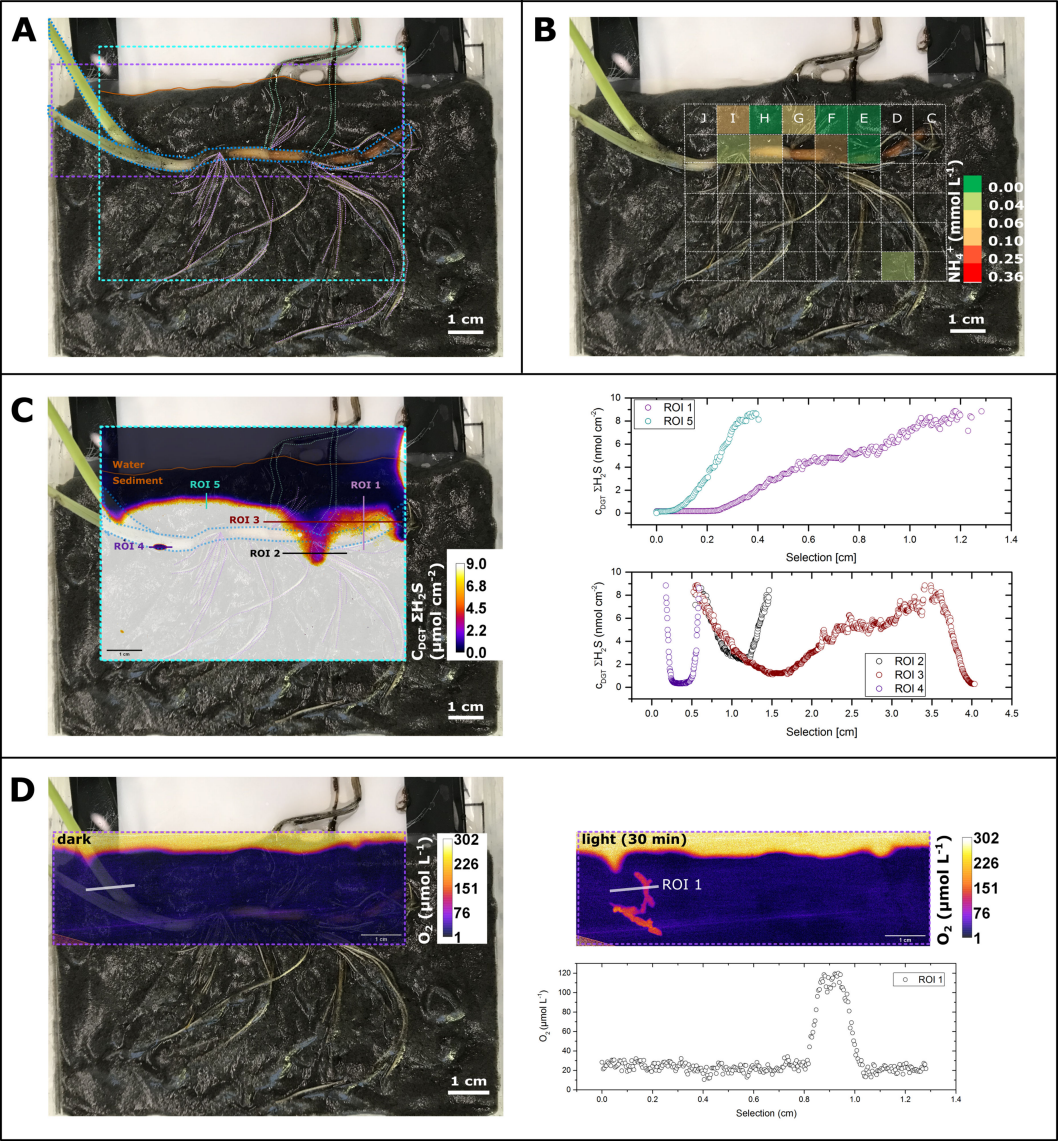
Distribution of O_2_, total sulfide, and ammonium concentrations in the seagrass rhizosphere. (**A**) Image shows the plant structures and position in the sediment. Dashed lines illustrate the position of the sensors. (**B**) Ammonium *c*_DET_ concentration around the below-ground seagrass tissue, where green indicates low and red high concentration. Transparent color code indicates values below the detection limit. The position of the DET gel is marked with the grid. (**C**) Total sulfide concentration around the below-ground seagrass tissue. Legend depicts the total *c*_DGT_ sulfide concentration, where transparent black indicates low and transparent white indicates high concentrations. Line profiles show the total sulfide concentrations across regions of interest (ROIs 1–5). The brown line marks the water-sediment interface. (**D**) Corresponding O_2_ concentration during light and dark conditions (after 30 min exposure time), with an extracted cross-tissue line profile (ROI 1). The oxygen image in panel D originates from Fig. 1.

**Fig 4:**
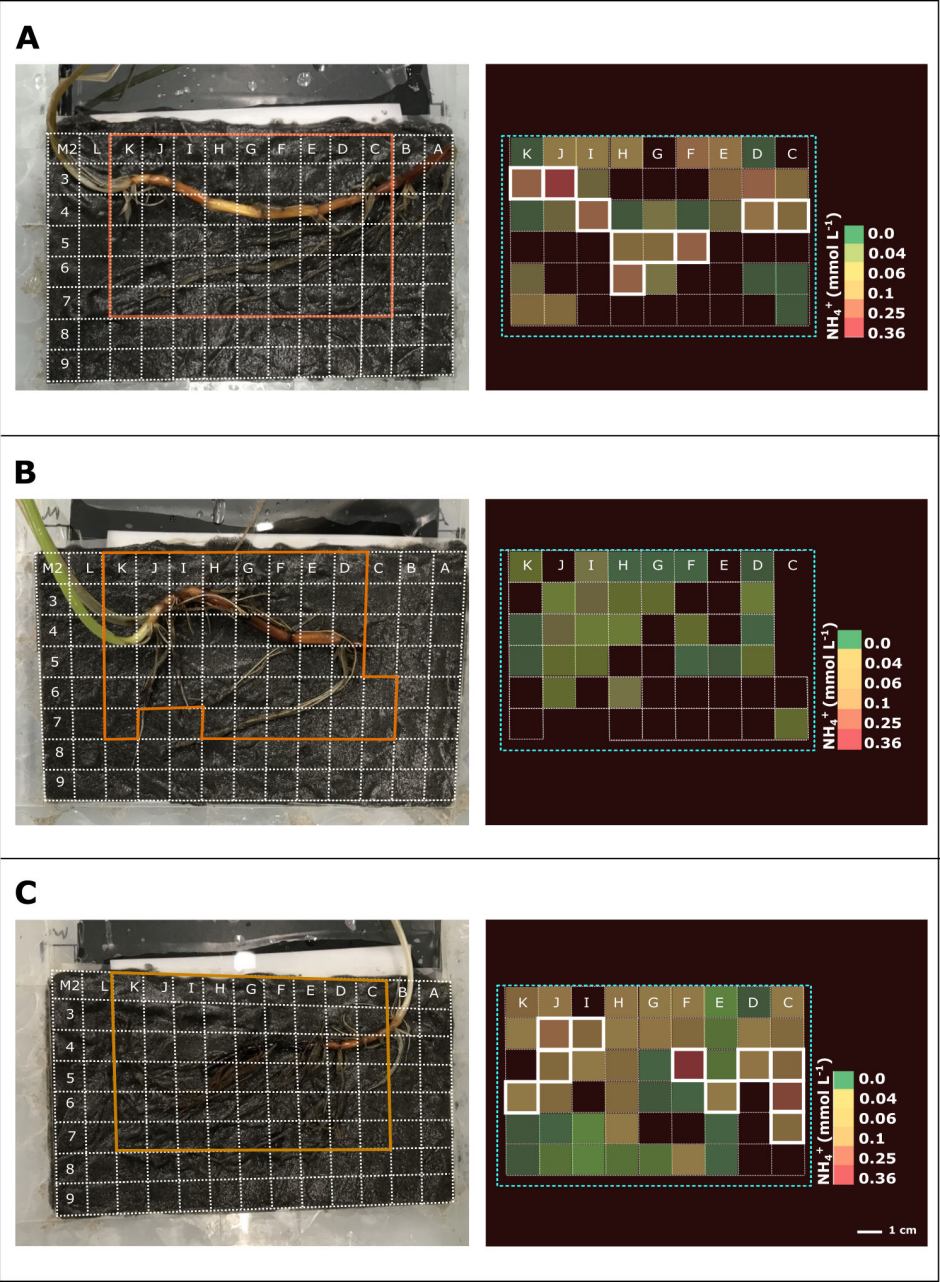
Ammonium concentration and distribution in the seagrass rhizosphere. Images on the left show the position of the *Z. marina* plant structures and the DET gels. Images on the right show the ammonium *c*_DET_ concentration around the below-ground tissue. The legend depicts the ammonium *c*_DET_ concentration, where green indicates low and red indicates high concentrations. Black color code indicates values below the detection limit. Panel A–C show plants 1–3; individual plants. Further biological replication can be found in Fig. **S8**. Sampled microhabitats for microbial community analysis are indicated with a white color code on the grid.

The ROL from below-ground tissue caused strong redox gradients in the seagrass rhizosphere, which resulted in heterogeneous sulfide oxidation (Fig. 3; Fig. S7) and thus local detoxification of this sediment-derived phytotoxin. Rhizosphere detoxification is especially important for young growing roots and the basal leaf meristems, as these lack barriers to the intrusion of phytotoxic gas (such as H_2_S) during elongation/growth (13, 58). In mature roots and rhizomes, gas-impermeable barriers consist of Casparian band-like structures composed of mainly suberin (58, 59) that ensure effective internal transport of O_2_ from above- to below-ground tissues of seagrasses (19), while also providing protection from sediment-produced reduced chemical compounds (13). Furthermore, we found relatively high concentrations of ammonium within these seagrass-mediated redox gradients in the rhizosphere, with NH_4_^+^ DET-concentrations (*c*_DET_) reaching up to ~400 µmol L^−1^ as compared to often not detectable NH_4_^+^ concentrations (*c*_DET_) of bulk seagrass sediment (Fig. 3 and 4; Fig. S8). Generally, high ammonium concentrations were found around the rhizome of the *Z. marina* plants, particularly around the basal leaf meristems and the rhizome nodes/root junctions (Fig. 4; Fig. S8), as well as along some regions of the mature part of the roots. Thus, ROL-derived oxidized sediment conditions might enhance the ammonium availability in the seagrass rhizosphere (Fig. 3), where heterogeneity results from multiple processes and fluxes including microbial metabolic processes such as N_2_-fixation, nitrification, and denitrification, as well as plant nitrogen uptake.

### Microbial community structure and diversity within rhizosphere chemical microhabitats

**Fig 5:**
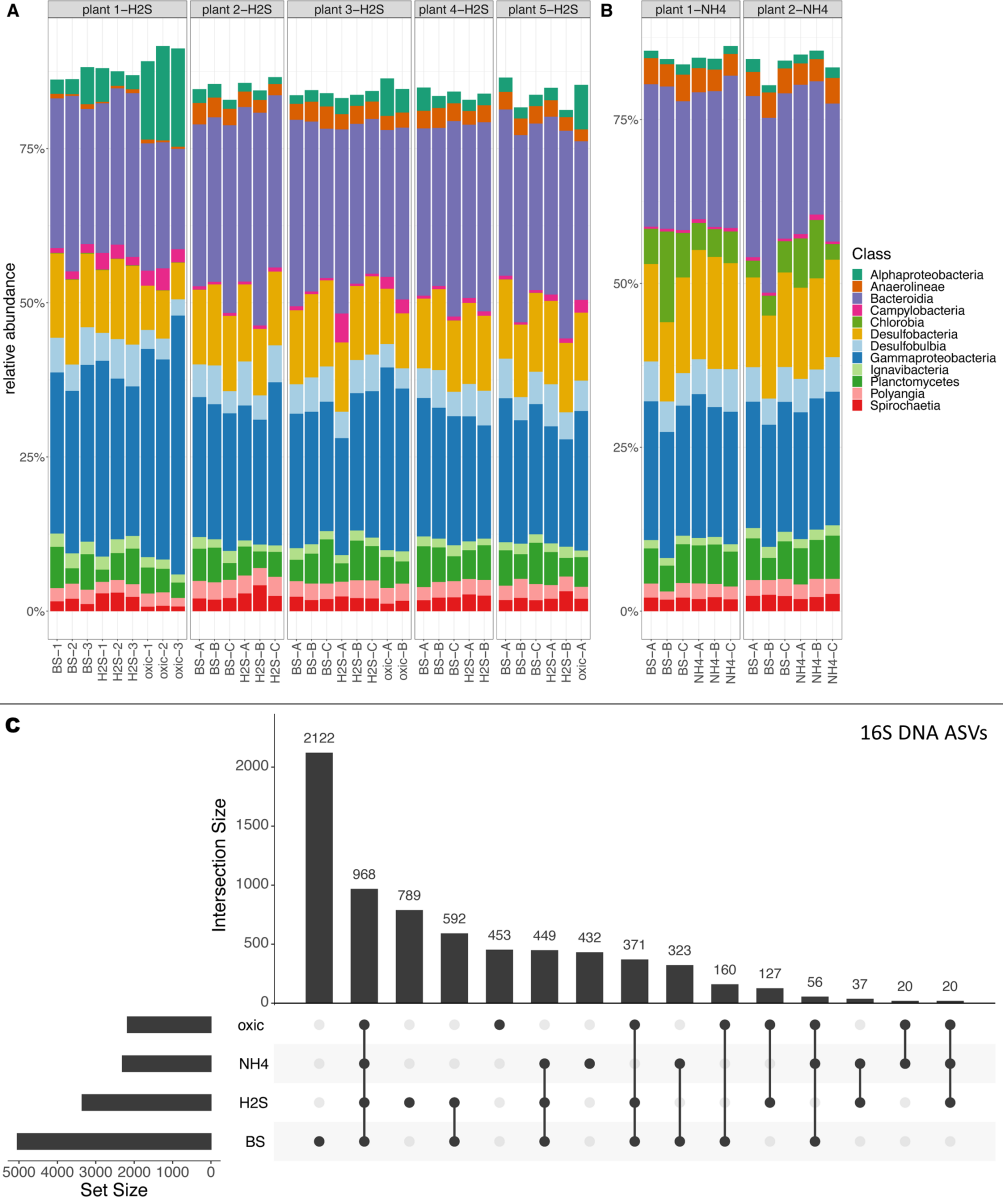
Microbial community composition within the seagrass rhizosphere. (**A–B**) Relative abundance of 16S rRNA gene amplicon ASVs of the most abundant 12 phylogenetic classes in the 16S data sets. The samples originate from distinct chemical sediment regions: BS = bulk sediment, H2S = high sulfide rhizosphere area, oxic = oxidized rhizosphere area, and NH4 = high NH_4_^+^ rhizosphere area. First set of measurements focused on the high sulfide areas around the *Z. marina* seagrass roots and rhizome (**A**). Second set of measurements targeted the high NH_4_^+^ areas of the seagrass rhizosphere (**B**). The top 12 classes accounted for >75% of the relative 16S gene abundance in all samples, with a core microbiome consisting of 13% of the taxa found across areas. (**C**) UpSet plot showing numbers of unique 16S DNA ASVs in the distinct chemical and microbial regions of the seagrass sediment.

We aligned sampling of the seagrass rhizosphere microbial community with the detailed mapping of the seagrass rhizosphere chemical microenvironment. This enabled us to identify distinct microbial communities in different chemical microhabitats (Fig. 5). Overall, the most abundant bacterial classes in the seagrass sediment were Bacteroidia (25%), Gammaproteobacteria (23%), Desulfobacteria (12%), and Desulfobulbia (5%) (Fig. 5A and B). However, the chemical microhabitats in the *Z. marina* rhizosphere selected for specific microbial communities with 789 unique amplicon sequence variants (ASVs) (i.e., 16S DNA ASVs; corresponding to 8.6% of the relative bacterial abundance) only present in the high sulfide areas, and 453 and 432 unique ASVs only present in the oxidized and high NH_4_^+^ rhizosphere areas, respectively (Fig. 5C). This indicates that distinct microbial communities are associated with the below-ground tissues of seagrasses, likely selected for by the local chemical conditions. Furthermore, we found a relative increase of Alphaproteobacteria in the oxidized rhizosphere areas (*P* = 8.5 × 10^−7^, Fig. 5A) as compared to BS, including the most abundant orders Rhodobacterales (3%), Rhodospirillales (6%), and Rhizobiales (1%). Rhizobiales includes plant symbionts like *Rhizobium* spp (52). Chlorobia was more abundant in samples from high NH_4_^+^ rhizosphere areas (*P* = 3.8 × 10^−7^, Fig. 5A and B). Chlorobia are sulfur oxidizers capable of anoxygenic photosynthesis in low light conditions, mainly found in the upper few millimeters of the sediment (60), and many members of this class are capable of N_2_ fixation (60).

**Fig 6:**
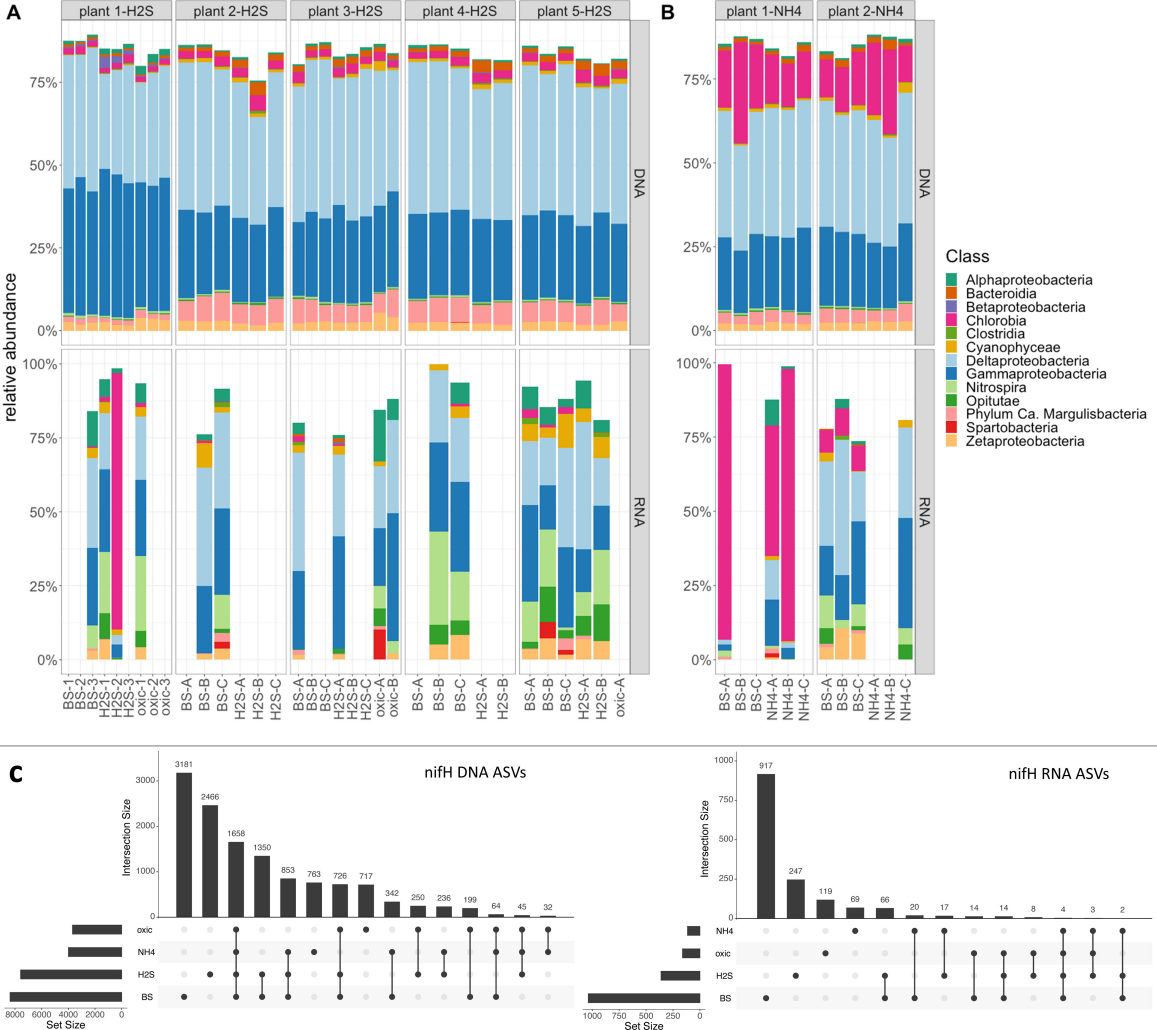
Diazotroph community composition associated with the seagrass rhizosphere. (**A–B**) Relative abundance of *nifH* DNA (top) and RNA (bottom) ASVs belonging to the 12 most abundant classes across the *nifH* data set. The *nifH* gene relative abundance and expressions of the key microbiome were determined within the sampled distinct sediment chemical regions: BS = bulk sediment, H2S = rhizosphere area with high sulfide concentration, oxic = oxidized rhizosphere area, and NH4 = rhizosphere area of high NH_4_^+^. First measurements focused on the high sulfide areas around the seagrass (*Z. marina*) roots and rhizome (**A**). Second set of measurements targeted high NH_4_^+^ areas of the seagrass rhizosphere (**B**). Note that *nifH* cDNA could not be amplified in all samples and some samples were removed in the processing of the sequences due to low sequencing depth (see Materials and Methods). (**C**) UpSet plots showing numbers of unique *nifH* DNA and RNA ASVs between the distinct chemical and microbial regions of the seagrass sediment.

Diazotrophs were widespread in the seagrass sediment (based on *nifH* DNA sequences), and 12% of the taxa were found associated to the chemical microhabitats in the seagrass rhizosphere (Fig. 6A and B; Fig. S9). Members of the candidate phylum Margulisbacteria were detected in relatively high abundances (5%) in the *nifH* DNA; they are proposed to be capable of H_2_ metabolism and fermentation (61). Delta- and Gammaproteobacteria dominated the *nifH* reads accounting for relative average abundances of 39% DNA (29% RNA) and 27% DNA (24% RNA), respectively (Fig. 6A and B). The main orders were Desulfobacterales and Desulfovibrionales indicating a prevalence of N_2_-fixing, SRB in the seagrass rhizosphere. This is consistent with earlier reports indicating a close relationship between seagrasses and N_2_-fixing SRB based on the reciprocal exchange of fixed carbon and dinitrogen (14, 16, 18). Additionally, *Nitrospira* accounted for 9% of the relative abundance in the *nifH* RNA sequences (Fig. 6A and B). *Nitrospira* is chemolithoautrotrophic bacteria that play an important role in biogeochemical nitrogen cycling by oxidizing nitrite to nitrate in the second step of nitrification (62, 63). Increased nitrate availability correlates with increased nitrate reductase activity in seagrass for nitrogen assimilation and protein production (64).

In the high sulfide rhizosphere areas, 2466 unique *nifH* DNA ASVs were found (Fig. 6C), indicating that this microenvironment harbors a distinct diazotrophic community. Nearly all active ASVs were unique to the seagrass-derived chemical microhabitats in the rhizosphere (Fig. 6 Fig. S10), with 247, 119, and 69 unique *nifH* RNA ASVs being present in the high H_2_S, oxidized and high NH_4_^+^ rhizosphere areas, respectively (Fig. 6C). Selection for specialized microbial communities in the rhizosphere microenvironments was further supported by an overall decreased alpha diversity within the seagrass rhizosphere, as compared to the BS (e.g., from ~6.1 to 5.7 in the *nifH* DNA alpha diversity index between BS and the high NH_4_^+^ rhizosphere areas [*P* < 0.05]; Fig. S11). The finding of unique *nifH* transcripts indicates that specific diazotrophs were active in the seagrass rhizosphere depending on the chemical condition of the microenvironments (i.e., oxic, high H_2_S, or NH_4_^+^ concentrations), and only a small number of ASVs were shared between the distinct chemical and microbial microhabitats (Fig. 6C).

### Rhizosphere chemical microenvironments for diazotrophic communities and activity

**Fig 7:**
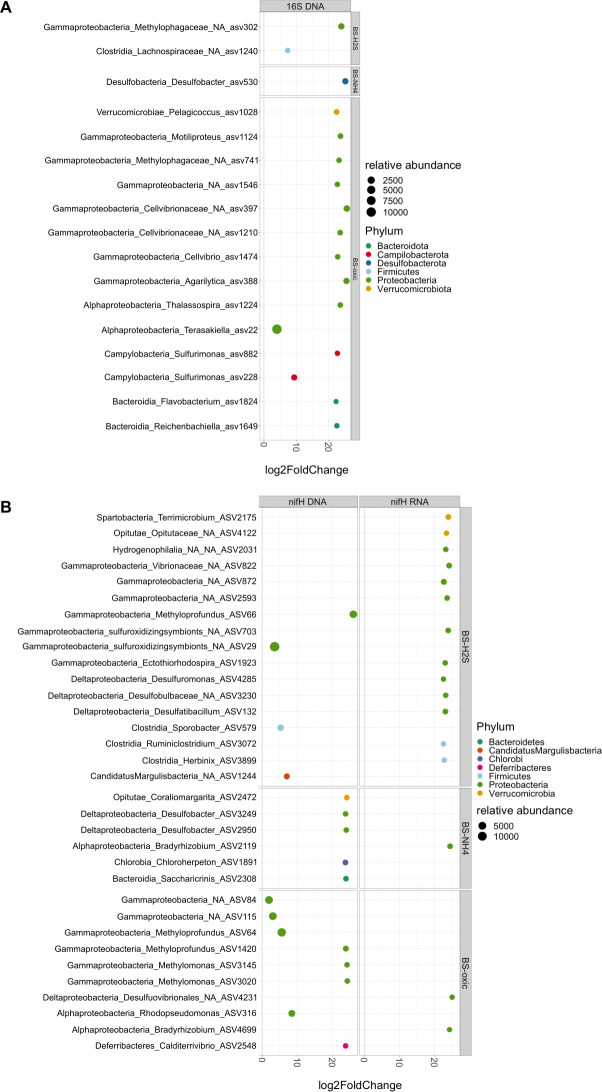
Differentially abundant 16S rRNA gene (**A**) and *nifH* RNA and DNA (**B**) ASVs between selected seagrass rhizosphere chemical regions as compared to the bulk sediment. Based on non-standardized ASV tables with read numbers, as the DESeq2 statistical model internally corrects for library size (65). Only significantly different ASVs shown (significance of *P* < 0.001) as determined by Wald test. Greater than zero log2 fold change indicates ASVs differentially more abundant within selected chemical regions of interest: H2S = high sulfide rhizosphere area, oxic = oxidized rhizosphere area, and NH4 = high NH_4_^+^ rhizosphere area.

Several functional groups of bacteria of putative benefit for the seagrass plant showed a higher differential abundance in chemical microenvironments within the seagrass rhizosphere, as compared with the microbial community composition in the BS (Fig. 7).

A 16S DNA ASV (16S rRNA gene) in the genus *Desulfobacter* exhibited a significantly higher abundance (*P*_adjust_ < 0.001) in the high NH_4_^+^ rhizosphere areas, as compared to the BS (Fig. 7A). *Desulfobacter* are SRB and many members of this genus can fix N_2_ (66, 67). These bacteria could thus provide bioavailable ammonium to the rhizosphere porewater. Two 16S DNA ASVs in the genus *Sulfurimonas* showed significantly higher relative abundance in oxidized rhizosphere areas relative to the BS (Fig. 7A). *Sulfurimonas* includes putative SOB that are important for seagrass sediment detoxification (11, 35, 68, 69), as bacterially mediated sulfide oxidation can be 1,000–10,000 times faster than chemical oxidation via O_2_ (70). *Sulfurimonas* could thus play an important ecological and functional role in the *Z. marina* rhizosphere by increasing the sulfide oxidation efficiency.

For the *nifH* DNA ASVs, methylotrophic bacteria showed a higher differential abundance in oxidized areas of the seagrass rhizosphere, as compared to the BS (Fig. 7B). Methylotrophic bacteria can promote plant growth and alleviate stress conditions by producing vitamins and phytohormones and can also fix N_2_, thereby potentially providing ammonium to *Z. marina* (71, 72). Methylotrophs are abundant in some aquatic plant rhizospheres, where they perform methane oxidation with plant released O_2_ (71, 72). *Methyloprofundus* and *Methylomonas* in the *Z. marina* rhizosphere (Fig. 7B), may thus be important for reducing methane emissions from seagrass sediments (73).

Analysis of *nifH* RNA ASVs showed a higher differential abundance and *nifH* gene expression of *Bradyrhizobium* in the high NH_4_^+^ and oxidized seagrass rhizosphere areas, as compared to the BS (Fig. 7B). We thus found co-localization of *Rhizobia nifH* gene expression and high rhizosphere ammonium concentrations. The genus *Bradyrhizobium* accounted on average for 5.3% of the relative *nifH* RNA abundance. This genus encompasses N_2_-fixing bacteria that often form symbiotic relationships with leguminous plants, where they fix N_2_ in exchange for carbohydrates from the plant (52–54). We speculate that a similar mutualistic relationship exists between the *Z. marina* plant and N_2_-fixing *Rhizobia* in the seagrass rhizosphere, as induced by seagrass-derived redox gradients. *Bradyrhizobium* was significantly more abundant in the oxidized part of the rhizosphere (*P* < 0.001), where it might contribute to ammonium accumulation around the seagrass below-ground tissue. Possibly through stimulation of Rhizobial growth and activity via plant-released organic acids, as well as ROL-driven alterations of sediment physiochemical conditions, such as slight rhizosphere acidification (e.g., through positive effects on nutrients solubility, and metals and reduced compounds toxicity) and protolytic dissolution of sediment-bound phosphates (34), which has been indicated to positively affect *Rhizobia* performance and thereby ammonium production (52–54). This may be vital for optimal seagrass performance and ecosystem functioning, especially in nitrogen-limited environments such as silicate-rich sediment in temperate coastal regions (48, 49).

*nifH* ASVs of Deltaproteobacteria (e.g., *Desulfuromonas* and *Desulfatibacillum*) and Clostridia (e.g., *Ruminiclostridium*) showed a higher differential abundance and *nifH* gene expression in rhizosphere areas with high sulfide concentrations (Fig. 7B). These bacteria include many sulfate or sulfur reducers that can fix N_2_ or produce ammonium via dissimilatory nitrate reduction (DNRA) (14, 38, 74). High sulfide concentration favors DNRA over denitrification owing to sulfide inhibition of the final two steps of denitrification (41). Thus, high but non-phytotoxic sulfide concentrations in regions of the rhizosphere may benefit the seagrasses by inducing the production of ammonium via DNRA. Interestingly, a Desulfobulbaceae ASV was found to be differently more abundant and active (based on *nifH* gene expression) in high sulfide rhizosphere areas, as compared to the BS (Fig. 7B). The family Desulfobulbaceae includes genera known as cable bacteria (e.g., *Candidatus* Electrothrix aarhusiensis) that are capable of coupling oxygen reduction with sulfide oxidation across centimeter-scale distances in the sediment (75–77). ROL-stimulated H_2_S oxidation by cable bacteria in the rhizosphere has recently been proposed to improve the sediment conditions enabling seagrasses to colonize and grow in sulfide-rich sediments (35, 77). Our molecular data thus support earlier observations showing that cable bacteria can be found in sulfidic areas of the rhizosphere (e.g., around root hairs) (76), where they colonize the rhizosphere at oxic–anoxic interfaces (35). Cable bacteria may also increase the nitrogen availability for seagrasses by indirectly promoting DNRA through iron sulfide dissolution (78) and/or via N_2_ fixation (75). However, in this study only a few 16S rRNA gene ASVs of the genus *Ca*. Electrothrix were detected and these were in low relative abundance.

### Conclusion

**Fig 8:**
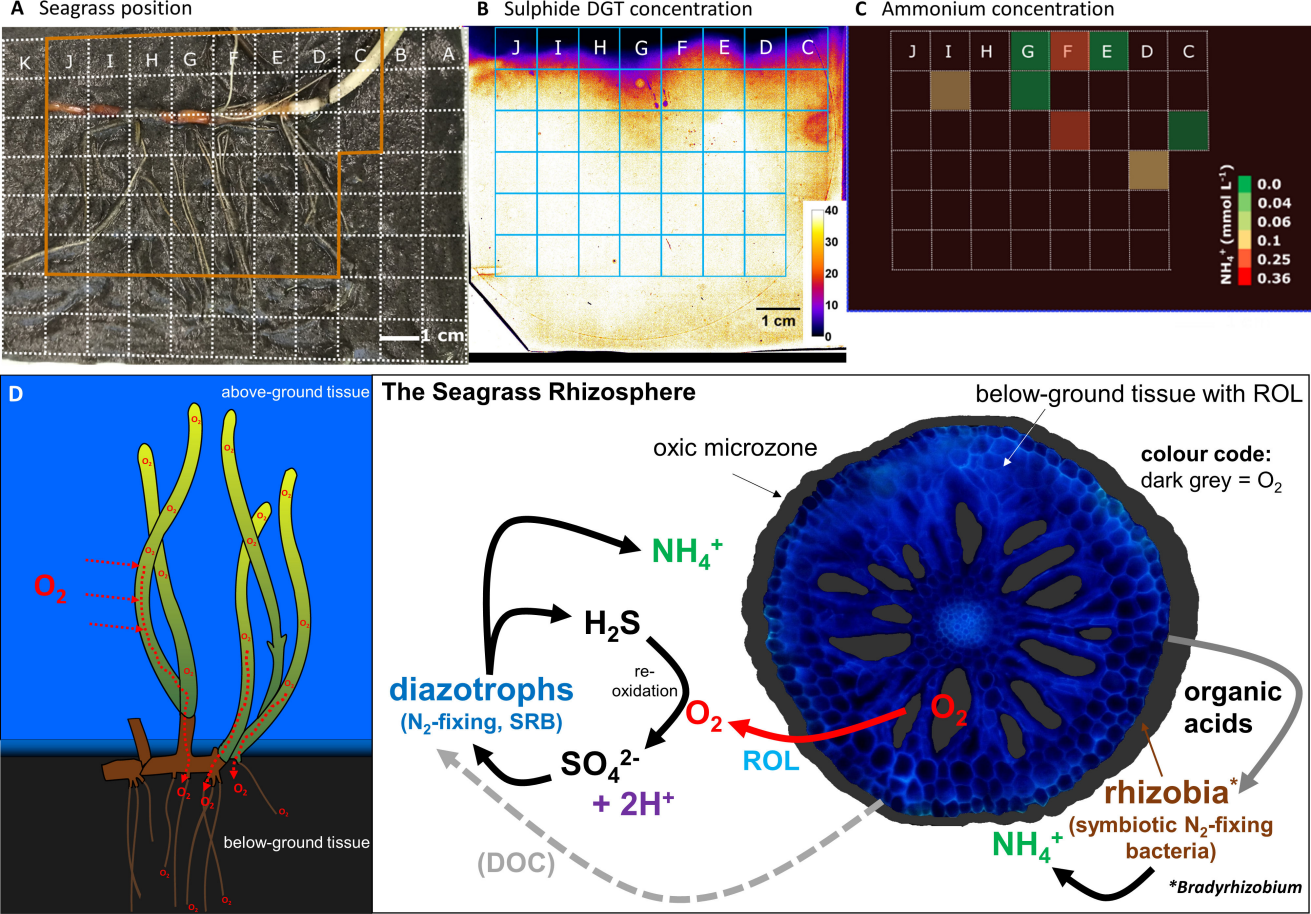
Conceptual summary of processes affecting ammonium levels in the rhizosphere. (**A–C**) Redox gradients around below-ground tissues of *Z. marina* seagrass are driven by radial O_2_ loss (ROL) and dissolved organic carbon (DOC) exudation from roots and rhizomes. This leads to ammonium accumulation in the rhizosphere. (**D**) Ammonium production within the seagrass rhizosphere is driven by multiple biogeochemical processes and chemical conditions, where diazotrophic *Rhizobia* (*Bradyrhizobium*) and sulfate-reducing bacteria are key microbial players.

Multiple biogeochemical pathways and microbial associations can lead to enhanced ammonium concentrations in the seagrass rhizosphere (Fig. 8), where especially N_2_-fixing, *Rhizobia* and SRB can be stimulated by the seagrass-mediated redox gradients. Our results indicate a strong association of abundant and active *Bradyrhizobium* in *Z. marina* rhizosphere regions with high porewater ammonium concentrations, indicating that *Rhizobia* function as growth-promoting rhizobacteria for seagrasses, especially in sediment environments with low bioavailable nitrogen levels. However, Deltaproteobacteria and Clostridia may also be important, as the general lower ammonium availability in high sulfide rhizosphere microhabitats often co-occurred with the position of the mature parts of the seagrass roots and therefore likely within rhizosphere regions with high rates of ammonium uptake through the root hairs. The release of O_2_ from *Z. marina* rhizomes and roots thus shapes the rhizosphere microbial community and serves numerous important ecological and biogeochemical functions for seagrass performance and health, which includes biotic and abiotic sediment detoxification, stimulation of nutrient solubilization, local production of ammonium, as well as likely promoting methane oxidation and, thus, potentially mitigating seagrass sediment greenhouse gas emissions.

## MATERIALS AND METHODS

### Sampling and experimental setup

*Z. marina* L. seagrass specimens and sediment were collected from a shallow coastal locality (<2 m depth) in North Zealand, Denmark (detailed description of Materials and Methods in Methods S1). Prior to measurements, the seagrass sediment was sieved to exclude infauna >1 mm. Twelve similar *Z. marina* plants were transplanted into the homogenized sediment in narrow, experimental “rhizobox” chambers with a removable transparent front window (34) enabling luminescence imaging from the front and gel-sampling-based mapping of sulfide and ammonium from the back of the chamber (further described below; Methods S1). The experimental chambers with seagrass, sediment, and sensor foils (*n* = 12) were positioned inside seawater aquaria (20°C, salinity of 18) in an upright position. Illumination of the seagrass leaves with a photon irradiance (PAR, 400–700 nm) of ~380 µmol photons m^−2^ s^−1^ was provided by MIA worldlight lamps (LP300Q-4K.24_RY; MIA Light GmbH, Gronau, Germany). Submerged water and air pumps ensured adequate flow and aeration of the seawater in the aquaria.

To enable gel sampling without disturbing the sediment, gels for single-analyte mapping of total sulfide (DGT gels) and ammonium (DET gels) concentrations (Methods S1) were positioned behind a fine, protective mesh (plankton mesh; DIN 100–60, mesh size 60 µm) (34). Single- and dual-analyte planar optode foils (i.e., for O_2_ and pH imaging; Methods S1) were mounted onto the removable front wall of the chamber. Seagrasses and sediment were left undisturbed within the experimental chambers for 48 h prior to chemical imaging, 96 h until gel imaging, and 168 h before sediment sampling for microbial analysis, to ensure measurements during steady-state biogeochemical/redox conditions (7, 34, 39, 79). To facilitate this, the sediment and the DGT/DET gels were separated by plastic foils preventing analyte diffusion into the gels before steady-state sediment biogeochemical conditions were reached (Methods S1) (34). Two sets of DGT/DET gels were deployed, thus enabling steady-state total sulfide and ammonium gel measurements in both darkness and light without disturbing the sediment during retrieval. To ensure steady-state light/dark conditions when measuring, a minimum of 5 h exposure before removal of the plastic foils was allowed (34), where after the gels were exposed to sediments and plants. This procedure enabled simultaneous steady-state measurements of aligned O_2_, total sulfide, and ammonium concentrations, as well as pH in the seagrass rhizospheres during light and dark conditions.

### Preparation of planar optical sensors and DET/DGT sampling gels

Planar optodes were prepared following published procedures (described in detail in Methods S1) (34, 80) using the O_2_ responsive luminescent dye Platinum(II)-meso(2,3,4,5,6-pentafluoro)phenyl-porphyrin (PtTFPP; Frontier Scientific, frontiersci.com) and the reference dye Macrolex fluorescence yellow 10GN (MY; KREMER, kremer-pigmente.de) with an optical isolation layer (excluding background fluorescence) consisting of carbon black (kremer-pigmente.de) knife-coated on top of the solid sensor film.

The dual pH and O_2_-sensitive planar optode was prepared according to Mosshammer et al. (80) by knife-coating three sensor layers consisting of: (i) an O_2_ sensitive layer (indicator dye: Eu(HPhN)_3_dpp; reference dye: Bu_3_Coum), (ii) a pH-sensitive layer (indicator dye: OHButoxy-aza-BODIPY, with diamond powder (microdiamant.com) serving as signal enhancer), and (iii) an optical isolation layer (carbon black and OHButoxy-aza-BODIPY) on top of dust-free PET support foil (Methods S1). Both single and dual analyte-sensitive planar optodes were calibrated using standard procedures (Methods S1, Fig. S1 and S2).

Sulfide-sensitive DGT gels were used for densitometric total sulfide mapping (Agl binding gels; Methods S1) (34). The sensor solution was knife-coated onto dust-free PET support foils using a PET foil as a spacer. DET gels were used for mapping ammonium concentrations (Methods S1) and were prepared according to published literature (81). Both the sulfide DGT and ammonium DET gels were calibrated using standard procedures (Methods S1, Fig. S3 and S4).

### Planar optode imaging and analysis

For O_2_ imaging, a ratiometric RGB camera setup (82) was used, while a 2CCD camera system (80) was used for simultaneous imaging of O_2_ and pH. Furthermore, details on the imaging systems are available in Methods S1. Acquired RGB color images were split into the red (R), green (G), and blue (B) channels and analyzed via the free-software ImageJ/fiji (rsbweb.nih.gov/ij/). Dissolved O_2_ concentration images were achieved by dividing the R channel (i.e., emission from the O_2_-sensitive indicator dye) of the color images with the G channel (i.e., emission from the inert reference dye) of the color images using the ImageJ/fiji plugin Ratio Plus (ratio = R/G). Subsequently, the obtained ratio images were fitted with the previously obtained calibration curve (Fig. S1) using the Curve Fitting tool of ImageJ/fiji (exponential decay function). The final zero value of the biological measurements was corrected to the red/green channel ratio acquired from the anoxic part of the sediment.

Acquired 2CCD images were split into the R, G, B, and near-infrared (NIR) channels and analyzed in ImageJ/fiji. For dissolved O_2_ concentration images, the R channel images (emission of the O_2_ sensitive Eu-complex) were divided by G channel images (emission from the coumarin reference dye) to calculate O_2_ dependent ratio images (ratio = R/G). The final zero value of the biological measurements were corrected to the red/green channel ratio acquired from the known anoxic part of the sediment. For pH distributions, the NIR channel images (emission of the pH-sensitive indicator dye: OHButoxy-aza-BODIPY) and the G channel images (emission of the coumarin reference dye) were divided to calculate pH dependent ratio images (ratio = NIR/G). Then, the obtained ratio images were fitted with the previously obtained calibration curves (Fig. S2) using an exponential decay function for O_2_ and a linear fit for the linear range of the sigmoidal pH response curve via the Curve Fitting tool of ImageJ/fiji. Calibrated O_2_ concentration and pH images were further analyzed in ImageJ.

### Sulfide DGT and ammonium DET gel measurements

The sulfide-binding AgI gels were deployed in the experimental chambers for 8 h during either light or dark exposure of the seagrass leaf canopy. Retrieved sulfide DGT gels were subjected to computer imaging densitometric (CID) analysis (34, 83): First, the protective mesh was removed from retrieved gels. The sulfide-sensitive gels were then fixed between two transparent PET foils, scanned at 600 dpi (Workcentre 7225, Xerox), and saved as color and greyscale TIFF files. The gels were left untreated, utilizing the contrast of the formed black Ag_2_S in the DGT gel against the pale white background at locations, where no sulfide was bound. The total sulfide distribution and concentration (S_tot_^2-^) in the experimental sample gels were then analyzed based on the previously acquired calibration function (Fig. S3).

The time-averaged S_tot_^2-^ concentration at the sampler-solution interface (i.e., *C*_DGT_ concentration of total sulfide) was calculated from the amount of S_tot_^2-^ accumulated during the total gel deployment time (83). This was done via CID analysis by quantifying the mass of S_tot_^2-^ taken up by the binding gel as a surface concentration *C*_s_ (µg cm^−2^) that was converted into the concentration at the sampler-exterior solution interface, *C*_DGT_, as:


$$C_{DGT}=C_{s}\frac{\Delta g}{Dt}$$

where *∆g* is the thickness of the diffusion layer overlying the AgI sulfide-binding gel, *D* is the S_tot_^2-^ diffusion coefficient inside the diffusion layer, and *t* is the gel deployment time. We note that the sulfide DGT measurement cannot be directly interpreted as an actual S_tot_^2-^ porewater concentration, as the sulfide DGT gel continuously binds S_tot_^2-^ from the exterior solution during deployment. The actual porewater concentration at the sampler-sediment interface is thus decreasing progressively during DGT gel sampling (81, 84).

The DET gels for ammonium determination were retrieved together with the other gels after 8 h of exposure. After retrieval, the gels were quickly cut into 1 × 1 cm squares, and treated according to the calibration procedure described in the Supporting Information (Methods S1, Fig. S4).

To facilitate precise DET-based ammonium determination and subsequent sediment sampling for molecular analysis within the pre-described distinct chemical microenvironments in the seagrass rhizosphere (i.e., oxidized, high sulfide, or high ammonium rhizosphere area), a detailed grid (1 cm^2^) system was made for the front and back chamber walls (Methods S1, Fig. S5).

### Molecular analyses

To characterize the microbial community, molecular analysis was carried out for the 16S rRNA gene and the diazotroph functional marker gene *nifH*. After the experiment, sediment was sampled from selected chemical microenvironments around the roots and rhizome of the seagrass, that is, BS, oxidized rhizosphere, high rhizosphere H_2_S, and high rhizosphere NH_4_^+^, as guided by the planar optode and DGT/DET measurements. Samples were flash frozen in liquid N_2_ and stored at −80°C. The first series of measurements (*n* = 8) focused on the oxic and high sulfide rhizosphere areas, the second series of measurements (*n* = 4) focused on the oxidized, and high ammonium rhizosphere areas.

#### Nucleic acid extraction

DNA was extracted from ~0.29 g sediment per sample using the DNeasy PowerSoil kit (Qiagen). RNA was extracted from ~2 g of sediment per sample with the RNA Power Soil kit (Qiagen). RNA was stored in multiple aliquots at −80°C. The RNA was reverse transcribed with the SuperScript IV First-Strand Synthesis System (Invitrogen, 10 U/µL) using a *nifH*3 reverse primer (0.2 µM final) in 10 µL reactions (85). Controls with RNase-free water instead of the reverse transcriptase were run to confirm the complete removal of DNA. Three amplicon libraries were generated for subsequent sequencing: 16S DNA, *nifH* DNA, and *nifH* cDNA. DNA amplicons for the V4/5 region of the 16S rRNA gene were generated with primer pair 515F-Y/926R (86). *nifH* gene amplicons were generated from DNA and cDNA with a nested PCR approach. The primer pair *nifH*3/*nifH*4 (87) was used in the outer PCR and the primers *nifH*1/*nifH*2 (88) with Illumina adapters were applied in the inner PCR. Approximately 5 ng of DNA was used in the PCR reactions with the KAPA HiFi Hot Start Ready Mix (Roche) and 0.2 µM of the respective primer pair for 16S and outer *nifH* DNA PCRs. For the cDNA, half of the cDNA reaction volume (5 µL) was used as a template for the outer *nifH* PCR reaction. For the inner *nifH* PCRs, 1–2 µL from the outer PCRs were used as a template (89). Triplicate reactions were pooled and cleaned using the Geneclean Turbo kit (MP Biomedicals, Germany). In a few cases, where *nifH* PCRs with cDNA showed unspecific amplification, amplicons of the correct size were cut from a 1% agarose gel (molecular grade) and purified with the PureLink Quick Gel Extraction Kit (Invitrogen). *nifH* cDNA amplicons could be generated for 34 of 53 samples. All amplicons were indexed in a 10 cycle PCR reaction, purified (Agencourt AMPure XP beads, Beckman Coulter), quantified (PicoGreen, Invitrogen), and pooled in equimolar ratios.

#### Amplicon sequencing and sequence analysis

Samples were sequenced with Illumina MiSeq (reagent kit v3, 2 × 300) at the GeoGenetics Sequencing Core (Copenhagen, Denmark). Raw demultiplexed paired-end sequences were processed into amplicon sequence variants (ASVs) using DADA2 (90) implemented in R-4.0.3. For *nifH* analysis, the parameters for “filterandTrim” were: truncLen = c(220,180), maxN = 0, maxEE = c(2,5), truncQ = 2, m.phix = TRUE, trimLeft = 17). Primer sequences were removed, reads were denoised, merged, and chimeric sequences removed (“mergePairs” and “removeBimeraDenovo”). Only sequences of 325–330 bp were kept. The generated ASVs were translated to amino acid sequences using FrameBot (91) and checked for non-target sequences by using the NifMAP pipeline (92). No non-target sequences were detected. Taxonomic ranks were assigned with DIAMOND blastp (93) using a FrameBot translated *nifH* database (94) based on the ARB database from the Zehr Lab (version June 2017; https://www.jzehrlab.com/nifh). Relationships to the canonical *nifH* clusters (95) were assigned according to Frank et al. (96) Four RNA samples that had less than 400 *nifH* reads were removed from the final data set as sequencing depth was considered too low. For 16S analysis, the parameters for “filterandTrim” were: truncLen = c(260,210), maxN = 0, maxEE = c(2,5), truncQ = 2, m.phix = TRUE, trimLeft = 20). Primer sequences were removed, reads were denoised, merged, and chimeras removed (“mergePairs” and “removeBimeraDenovo”). Only sequences of 369–375 bp were kept. Read annotation was achieved with the function ‘assignTaxonomy’ using the SILVA database (Silva v138.1).

Furthermore, analyses were performed in R (R Core Team 2022, 4.1.3) with the R package phyloseq (v. 1.38.0) (97) for handling sequence abundance tables. Chloroplast and mitochondria sequences were removed from the 16S data set (Table S1). Sequence abundance tables were normalized to median sequencing depth. To estimate alpha diversity, the Shannon index was calculated with the “estimate_richness” function of phyloseq. The Wilcoxon test was used to test for statistical differences in alpha diversity and relative abundances. Singletons were excluded from further analysis. The differential abundance analysis was carried out in DESeq2 (65) on non-standardized reads data, as the DESeq2 statistical model internally corrects for library size, by comparing areas of distinct chemical microenvironments (i.e., H_2_S, NH_4_^+^, oxic) to the BS. ASVs with less than 50 reads in total were filtered out in each data set. First, the size factors were estimated by applying “poscounts” as it accounts for ASVs missing in some samples and the setting “local” was then used for the dispersion estimate. The Wald test and the adjusted *P* value of <0.001 were applied to test for significance. UpSet plots were produced with UpSetR (v1.4.0) (98), other plots were generated using ggplot2 (v3.3.3) (99) and ggpubr (v0.4.0) (100).

## Data Availability

All the original contributions presented in the study are included in the article/supplementary material. Sequences are deposited in Sequence Read Archive under project accession number PRJNA988287. Furthermore, inquiries can be directed to the corresponding author (K.E.B.).

## SUPPLEMENTAL MATERIAL

Methods S1, Figures S1-S11, and Table S1. spectrum.03335-23-s0001.docx Supplementary information.

ASM does not own the copyrights to Supplemental Material that may be linked to, or accessed through, an article. The authors have granted ASM a non-exclusive, world-wide license to publish the Supplemental Material files. Please contact the corresponding author directly for reuse.
