# Supplementary material for: Seagrass-mediated rhizosphere redox gradients are linked with ammonium accumulation driven by diazotrophs
Source: Microbiol Spectr. 2024 Mar 1;12(4):e03335-23. doi: 10.1128/spectrum.03335-23 (PMC10986515; doi:10.1128/spectrum.03335-23)
Supplement: Methods S1, Figures S1-S11, and Table S1. — Supplementary information. [file spectrum.03335-23-s0001.docx]

**SUPPLEMENTARY INFORMATION**

**Seagrass-mediated rhizosphere redox gradients are linked with ammonium accumulation driven by diazotrophs**

Kasper Elgetti Brodersen^1, *,#^, Maria Mosshammer^1,*^, Meriel J. Bittner^1,*^, Søren Hallstrøm^1^, Jakob Santner^2^, Lasse Riemann^1^, Michael Kühl^1^

^1^Marine Biological Section, Department of Biology, University of Copenhagen, Helsingør, Denmark.

^2^Department of Crop Sciences, Institute of Agronomy, University of Natural Resources and Life Sciences Vienna, Tulln an der Donau, Austria.

^*^These authors contributed equally to this study and therefore share the first authorship of the manuscript.

^#^Corresponding author: Kasper Elgetti Brodersen ([elgetti@ruc.dk](mailto:elgetti@ruc.dk))

Present address: Environmental Dynamics, Department of Science and Environment, Roskilde University, 4000 Roskilde, Denmark.

*Running title:* Seagrass rhizosphere nitrogen mobilisation

*Contents:* Methods S1, Figures S1-S11, and Table S1.

*Supporting Figures:*

**Fig. S1.** O_2_ sensor calibration (planar optode with ratiometric SLR camera read-out).

**Fig. S2.** Dual O_2_ and pH sensor calibration (planar optode with ratiometric 2CCD camera read-out).

**Fig. S3.** Total sulphide (S_tot_^2-^) sensor calibration (DGT gel).

**Fig. S4.** NH_4_^+^ sensor calibration (DET gel).

**Fig. S5.** Experimental setup, chamber design and suite of sensors.

**Fig. S6.** O_2_ and pH distribution in the seagrass rhizosphere – plant 2.

**Fig. S7.** O_2_, pH and total sulphide distribution in the seagrass rhizosphere – plant 3.

**Fig. S8.** NH_4_^+^ concentration and distribution in the seagrass rhizosphere – plants 5-8.

**Fig. S9.** Relative abundance of nifH DNA and RNA amplicon ASVs.

**Fig. S10.** Relative abundance of the 16S and nifH unique ASVs for the selected regions of interest: bulk sediment, oxidized rhizosphere area, high rhizosphere H_2_S and high rhizosphere NH_4_^+^.

**Fig. S11.** Alpha diversity of the 16S rRNA gene estimated by the Shannon diversity index.

**Table of Contents**

**Materials and Methods:**

1. Seagrass and sediment sampling
2. Preparation of optical sensors
3. Preparation of diffusive gradients and equilibrium in thin films (DGT and DET) gels
4. Camera systems and setups for optical sensor signal read-out
5. Planar optode calibration
6. Luminescence imaging and analysis
7. Sulphide DGT and ammonium DET gels calibration
8. DGT and DET analysis and interpretation
9. Experimental Setup
10. Nucleic acid sampling, extractions and amplicon sequencing
11. Sequence processing and taxonomic annotation
12. Molecular data analysis

**Supplementary Data:**

1. Figs. S6 and S7: O_2_, pH and total sulphide concentration and distribution – plant 2 and 3.
2. Fig. S8: Ammonium concentration and distribution – plants 5-8.
3. Fig. S9: nifH community composition subclusters.
4. Fig. S10: 16S and nifH unique ASVs.
5. Fig. S11: Shannon diversity index.
6. Table S1: Number of ASVs after each cleaning step.

**References**

**Methods S1**

**MATERIALS AND METHODS**

*(a) Seagrass and sediment sampling*

Specimens of the seagrass *Zostera marina* L. and sediment were collected from a shallow coastal site (<2m depth) at Julebæk, North Zealand, Denmark (56°03'29.2"N; 12°34'40.7"E) during the autumn of 2019. After sampling, seagrass and sediment were kept in a large seawater reservoir that was continuously thermostated and aerated (20°C; salinity of 18), and illuminated by metal-halide lamps (Megachrome, Giesemann Aquaristic GmbH, Nettal, Germany) at an incident photon irradiance (PAR, 400-700 nm) of ~200 µmol photons m^-2^ s^-1^ in a 14h:10h light/dark cycle. Thus, mimicking the environmental conditions at the sampling site. Twelve similar *Z. marina* specimens were selected from the reservoir for the experiment (n = 12) and were planted in sediment, which was sieved to obtain the <1 mm grain size fraction and thus exclude larger infauna.

*(b) Preparation of optical sensors*

Dual and single analyte-sensitive planar optodes were prepared by knife-coating sensor cocktails onto transparent polyethylene terephthalate (PET) support foils (goodfellow.com) as follows (e.g., Mosshammer et al. 2016; Brodersen et al. 2017). The sensor cocktail used for the planar O_2_-sensitive optodes consisted of 1.5 mg of Platinum(II)-meso(2,3,4,5,6-pentafluoro)phenyl-porphyrin (PtTFPP; Frontier Scientific, frontiersci.com), 1.5 mg of Macrolex® fluorescence yellow 10GN (MY; KREMER, kremer-pigmente.de), 100 mg of polystyrene (PS, MW 250.000 g mol^-1^; ACROS Organics, across.com) dissolved in 1 g of THF (Sigma-Aldrich, sigmaaldrich.com). The cocktail was spread onto dust-free PET foils using a knife coater (byk.com) to yield a ~10 µm thick optical sensor film after solvent evaporation. An optical isolation layer consisting of 1% w/w (10 mg) of carbon black (kremer-pigmente.de) dispersed in 1 g 10% w/w solution (EtOH: water, 9:1 w/w) of polyurethane hydrogel (Hydromed D4; AdvanSource Biomaterials, advbiomaterials.com) was then knife-coated on top of the sensor film (thickness of ~7.5 µm) to exclude background fluorescence and light scattering from the sediment, and thus optimize the spatial resolution of the planar optode.

The applied dual pH and O_2_-sensitive planar optode was prepared by knife-coating three sensor layers consisting of first an O_2_ sensitive layer, then a pH sensitive layer and finally an optical isolation layer on top of dust-free PET support foils (Mosshammer et al. 2016). The sensor solution for the O_2_-sensitive layer consisted of 2 mg of Eu(HPhN)_3_dpp (indicator dye) and 0.3 mg of Bu_3_Coum (reference dye; both synthesized according to Mosshammer et al. 2016) dissolved in 3.33 g 12% w/w solution of PS (in toluene). The sensor cocktail was knife-coated onto the PET films resulting in a ~2.5 µm thick layer after solvent evaporation. The sensor solution for the pH sensitive layer consisted of 0.05% w/w (1 mg) of OHButoxy-aza-BODIPY (indicator dye; synthesized according to Mosshammer et al. 2016) dissolved in 250 µL THF, added to 2 g 10% w/w solution of D4 (isopropanol:water, 9:1 w/w) containing 10% w/w (200 mg) diamond powder (microdiamant.com), serving as a sensor signal enhancer. The sensor cocktail was knife-coated on top of the O_2_ sensitive layer yielding a ~7.5 µm thick pH sensitive layer after solvent evaporation. The chemical solution for the optical isolation layer consisted of 1% w/w (20 mg) of carbon black dispersed in 2 g 10% w/w solution of D4 (isopropanol:water, 9:1 w/w) and 0.05% w/w (1 mg) of OHButoxy-aza-BODIPY dissolved in 250 µL THF. The optical isolation layer was knife-coated on top of the pH sensitive layer yielding a ~7.5 µm thick optical isolation layer after solvent evaporation. The final dual-analyte-sensitive planar optode with optical isolation had thus a total thickness of <20 µm (Mosshammer et al. 2016).

*(c) Preparation of diffusive gradients and equilibrium in thin films (DGT and DET) gels*

Agl binding gels for densitometric total sulphide mapping (i.e., sulphide DGT gels) consisted of 5 g of Agl dispersed for 5 minutes in a 30 g 10% w/w solution of D4 (ethanol:water, 9:1 w/w) using an Ultra-Turrax disperser (Brodersen et al. 2017). This solution was then knife-coated onto dust-free PET support foils using a PET foil (0.125 mm thickness) as spacer (Brodersen et al. 2017).

Diffusive equilibrium in thin film (DET) gels were used for mapping of ammonium availability. The gels were prepared according to standard procedures (Zhang et al. 1999). In short: 15 mL of DGT gel cross-linker were added to 47.5 mL MilliQ water. 37.5 mL of a 40% w/w acrylamide solution were added and everything was mixed together. This stock solution was kept at 4 °C until use. 2 mL of this solution were mixed with 14 µL ammonium persulfate (APS) solution (10% w/w) and 5 µL N, N, N’, N’ Tetramethylethylene-1,2-diamine (TEMED) and immediately pipetted in a glass chamber, consisting of two acid-washed glass plates separated by a 0.5 mm spacer. The chamber was placed in an oven at 42 °C for 40 min for polymerization. The gel was retrieved from the chamber, soaked in MilliQ for 24 h, with at least three water changes, and subsequently stored in a NaCl solution.

*(d) Camera systems and setups for optical sensor signal read-out*

For O_2_ imaging, a custom-made ratiometric (RGB) camera setup was used consisting of a Canon SLR camera (EOS 1000D, Canon, Japan) equipped with a macro-objective lens (Macro 100 f2, 8 D, Tokina, Japan) that had a 530 nm long-pass filter (uqgoptics.com) and additionally a plastic filter (#10 medium yellow; leefilters.com) mounted in front of the macro-objective lens to reduce the background fluorescence. Excitation of the planar O_2_-sensitive optode was achieved via a 455 nm multichip LED (LedEngin Inc, RS Components Ltd, Corby, UK), that was controlled and powered by a USB-controlled LED driver unit (imaging.fish-n-chips.de), combined with a bandpass filter. Image acquisition and control of the SLR camera and the LED driver unit were achieved with the software look@RGB (imaging.fish-n-chips.de).

For dual O_2_ and pH imaging, a similar ratiometric camera approach was used (Mosshammer et al. 2016). However, here consisting of a 2CCD multispectral camera (JAI AD-080 GE; jai.com) equipped with a Schneider KREUZNACH video objective lens (1.4/23 CCTV-LENS 400-1000 nm; schneiderkreuznach.com) mounted with a 510 nm long-pass filter (OG 510 Schott) in combination with an additional plastic filter (#10 medium yellow; leefilters.com) to reduce background fluorescence. A high-power 405 nm LED (LedEngin; rs-online.com) with a custom-built LED trigger system (National instruments USB 6008) was used as excitation source. Image acquisition and control of the LED and 2CCD camera were achieved via the custom-made software from Bioras Aps (bioras.com). Camera settings for the RGB chip were 0.8s exposure time and a receiver gain of 30. For the near-infrared (NIR) chip the exposure time was 0.8s and the receiver gain 75.

*(e) Planar optode calibration*

The O_2_-sensitive planar optodes were calibrated by mounting a planar O_2_ optode on the inside of the front glass wall of the narrow, experimental chamber (described in detail below). The chamber was then filled with seawater (20°C, salinity of 18) wherein the O_2_ concentration was altered via compressed nitrogen and air mixed by a PC-controlled gas mixer (SensorSense, The Netherlands). The dissolved O_2_ concentration inside the chamber was monitored using a fiber-optic O_2_ meter (Firesting GO_2_, PyroScience GmbH, Aachen, Germany). To ensure that steady-state O_2_ conditions were reached, each calibration step was held for a minimum of 5 min before an image was acquired. The final O_2_ calibration of the single-analyte planar optode was obtained from the acquired RGB images by relating the extracted red (R)/green (G) channel ratios to the actually measured dissolved O_2_ concentration (Fig. S1).


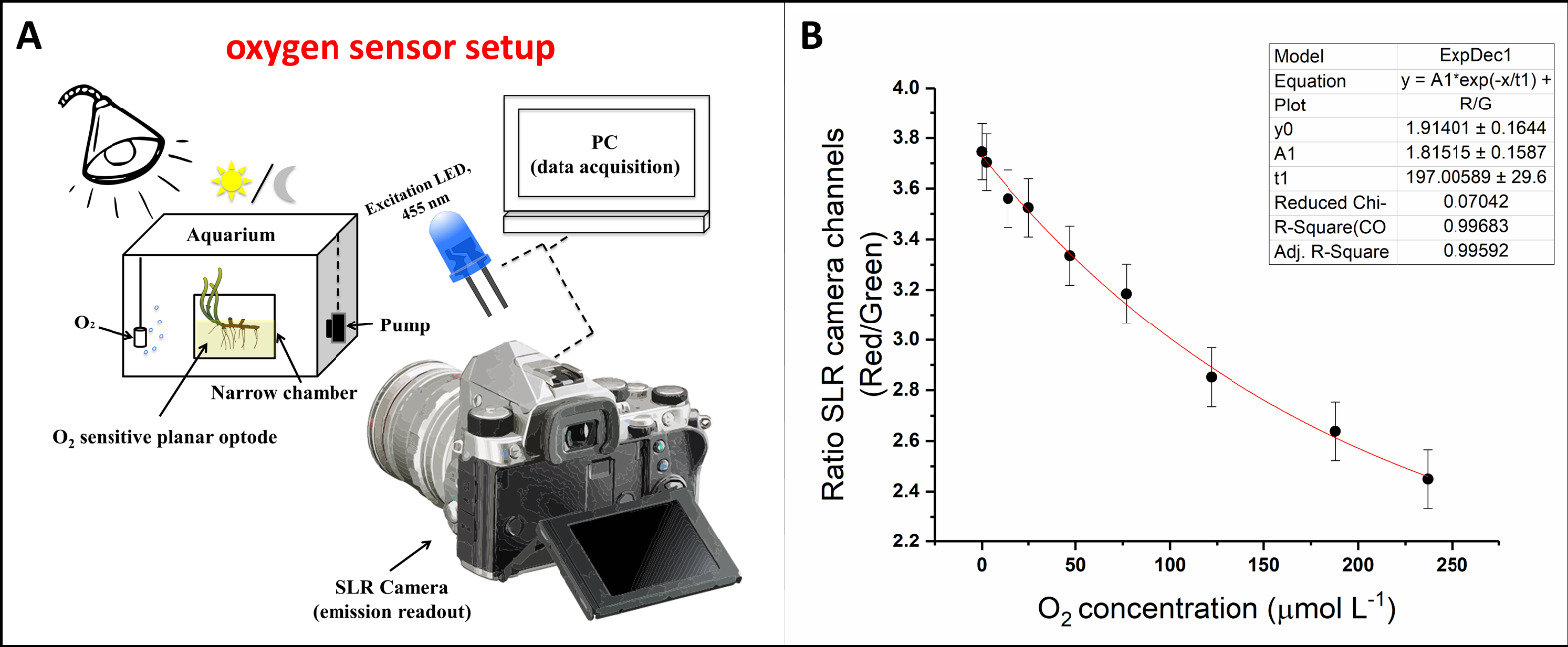


**Figure S1.** Single-analyte planar optode system (A) and calibration (B). The mean R/G image ratios were fitted with an exponential decay function (R^2^ = 0.99).

The pH and O_2_ sensitive planar optode was calibrated in a similar manner, where the calibration curve for pH was generated by exposing the dual-analyte planar optode to buffer solutions of known pH (pH of 3.7, 6.7, 7, 7.5, 7.9, 8.7, 9 and 10.1) as measured using a calibrated pH meter (PHM 220, Radiometer Copenhagen, Denmark); with the salinity adjusted to 18 using NaCl, as measured with a calibrated refractometer. The final O_2_ and pH calibrations were obtained by relating the extracted R/G channel ratios to the measured dissolved O_2_ concentrations and the NIR/G channel ratios to the known pH values, respectively (Fig. S2).

**
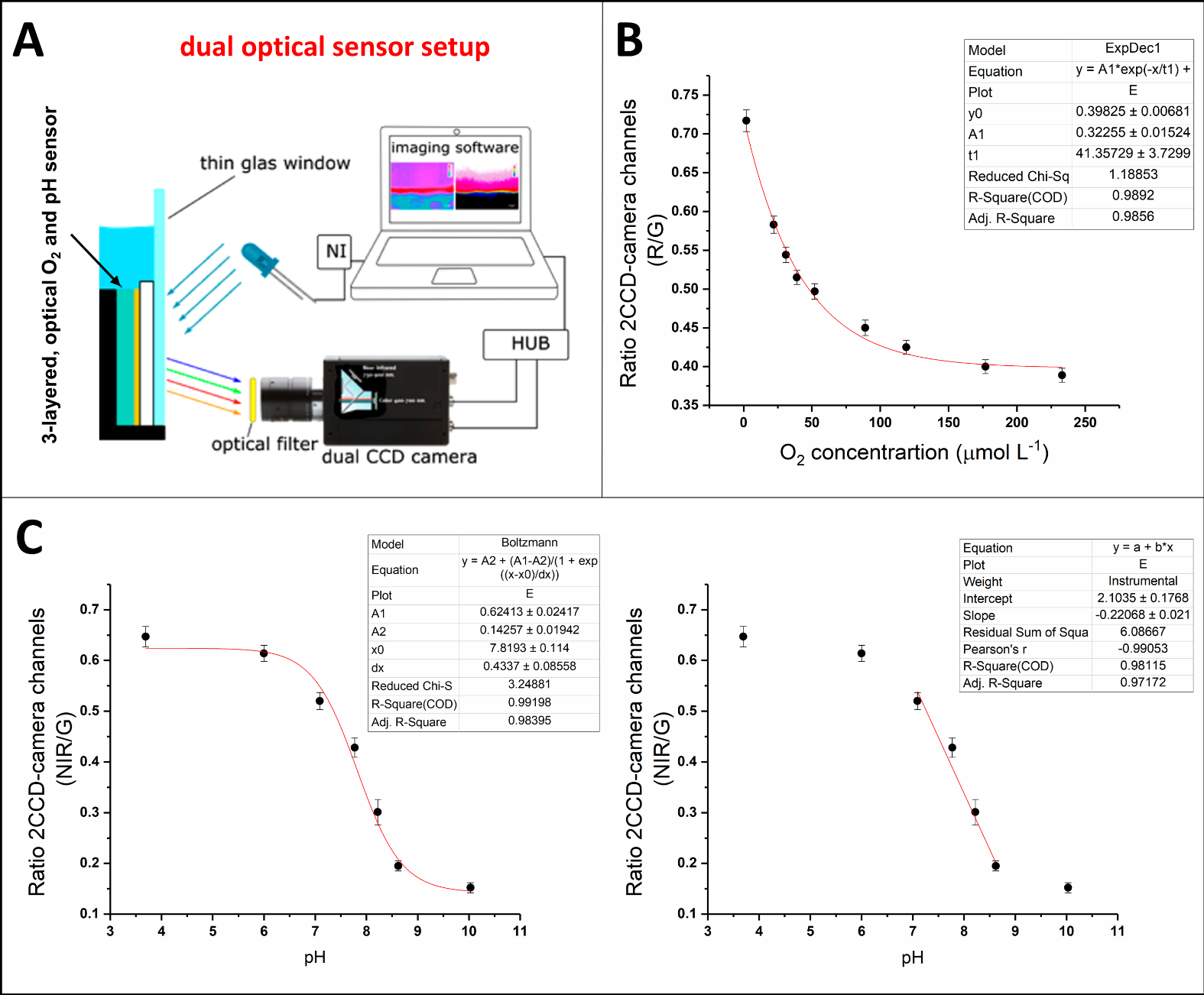
**

**Figure S2.** Dual-analyte planar optode system (A), calibration of the O_2_-sensitive layer (B) and calibration of the pH-sensitive layer (C). The mean R/G image ratios were fitted with an exponential decay function (R^2^ = 0.99) for O_2_ determination and the mean NIR/G image ratio with a sigmoid (left) and linear (right) function for pH determination (R_2_ = 0.99 and 0.98, respectively). Panel A, is partly redrawn from Mosshammer et al. 2016.

*(f) Luminescence imaging and analysis*

For the single-analyte planar O_2_ optode, the acquired RGB colour images were split into the red, green and blue channels and analysed via the free-software ImageJ/fiji (rsbweb.nih.gov/ij/). Dissolved O_2_ concentration images were achieved by dividing the red channel (i.e., emission from the O_2_-sensitive indicator dye) of the colour images with the green channel (i.e., emission from the inert reference dye) of the colour images using the ImageJ/fiji plugin Ratio Plus (ratio = R/G). Subsequently, the obtained ratio images were fitted with the previously obtained calibration curve (Fig. S1) using the Curve Fitting tool of ImageJ/fiji (exponential decay function). The final zero value of the biological measurements were corrected to the red/green channel ratio acquired from the anoxic part of the sediment.

For the dual-sensitive planar optode, the acquired images were split into the red (R), green (G), blue (B) and near-infrared (NIR) channels and analysed likewise via ImageJ/fiji. For dissolved O_2_ concentration images, the red channel images (emission of the O_2_ sensitive Eu-complex) were divided by green channel images (emission from the coumarin reference dye) as ratio = red/green. The final zero value of the biological measurements were corrected to the red/green channel ratio acquired from the known anoxic part of the sediment. For pH distributions, the NIR channel images (emission of the pH sensitive indicator dye: OHButoxy-aza-BODIPY) and the green channel images (emission of the coumarin reference dye) were divided (ratio = NIR/G). Then, the obtained ratio images were fitted with the previously obtained calibration curves (Fig. S2) using an exponential decay function for O_2_ and a linear function for pH via the Curve Fitting tool of ImageJ/fiji. Calibrated O_2_ concentration and pH images were further analysed via ImageJ.

*(g) Sulphide DGT and ammonium DET gels calibration*

The sulphide-binding AgI gels were calibrated by exposing a defined surface area of the sulphide DGT gels to known total sulphide (S_tot_^2-^) concentrations (i.e., 0, 50, 100, 250, 500, 750, 1000, 2500, 5000 and 1000 µmol S_tot_^2-^ L^-1^) over a given time period (here, 8 h exposure). S_tot_^2-^ stock solutions (0.01M and 0.05M) were prepared by dissolving known amounts of Na_2_S in deoxygenated miliQ water. Afterwards, retrieved gels were subjected to computer imaging densitometric (CID) analyses, conducted by using a printer with incorporated flatbed scanner (Workcentre 7225, Xerox). From the obtained grayscale values and the known S_tot_^2-^ DGT-concentrations (c_DGT_), calibrations functions were determined using ImageJ (readout of grayscale values) and Origin Pro (data analysis and fitting; OriginLab Corp., USA). The applied S_tot_^2-^ calibration curve is provided below (Fig. S3). Determined amounts are referred to as c_DGT_, as they are time-dependent accumulative ‘concentrations’ and not real concentrations.

**
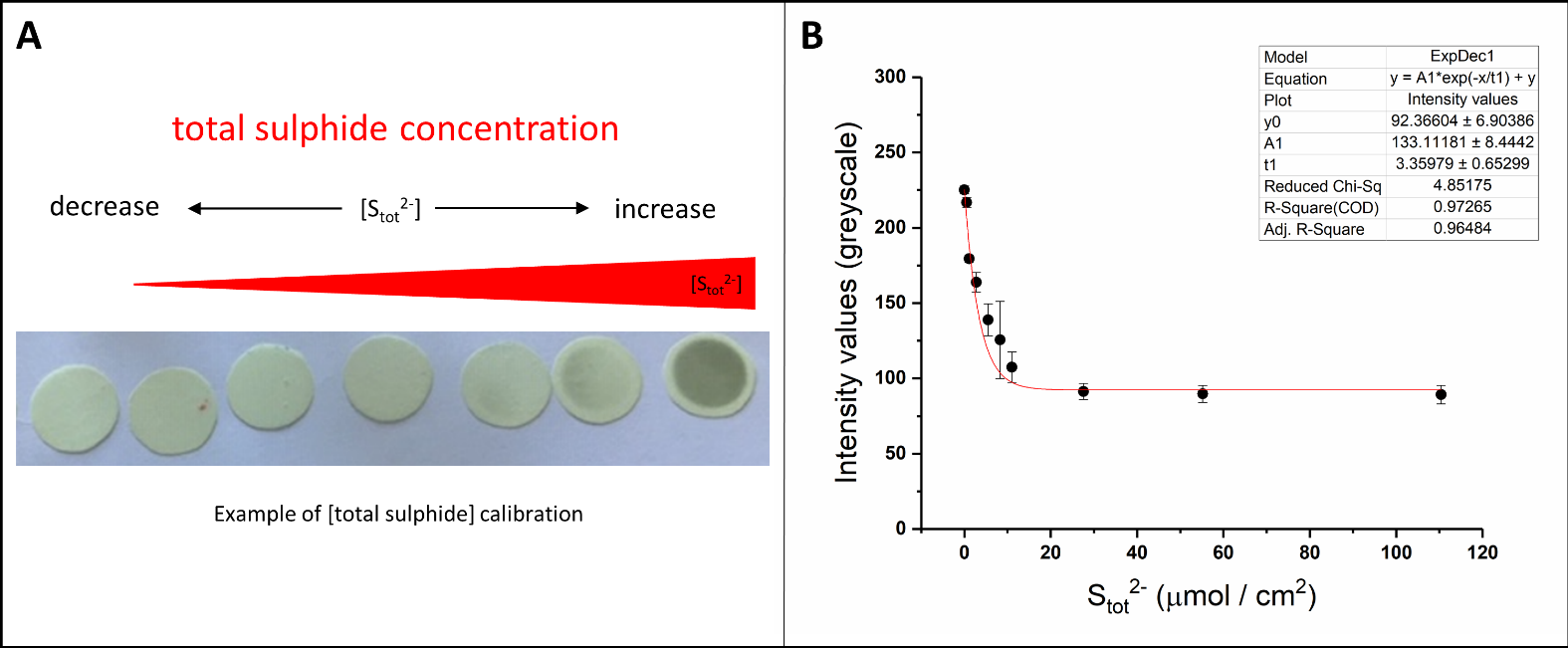
**

**Figure S3.** Example of sulphide-induced colouration of AgI binding gels (i.e., sulphide sensitive DGTs) at increasing total sulphide concentration (A) and the mean greyscale intensity values of the actual sulphide DGT gel calibration at increasing total sulphide concentration (B) fitted with an exponential decay function (R^2^ = 0.97).

The ammonium equilibrium gels were calibrated by exposing precisely cut squares (1 x 1 cm) of the DET gels between pieces of the used filter (polycarbonate, 0.22 Micron, GE Water & Process Technology,) to solutions with known concentrations of ammonium (Fig. S4). A 10 mM solution of NH_4_Cl was used for preparation of the calibration solutions. The gels were soaked in 10 mL of the calibration solutions for 5 hours, to ensure equilibrium. The gels were retrieved and placed in 1 mL MilliQ water each, for 24 h to extract the dissolved ammonium. These solutions were then analysed using ammonium test kits from Spectroquant®, purchased from Merck (https://www.sigmaaldrich.com/; 1.00683 and 1.14752) for photometric analysis (UV-1800 spectrophotometer, Shimadzu).

**
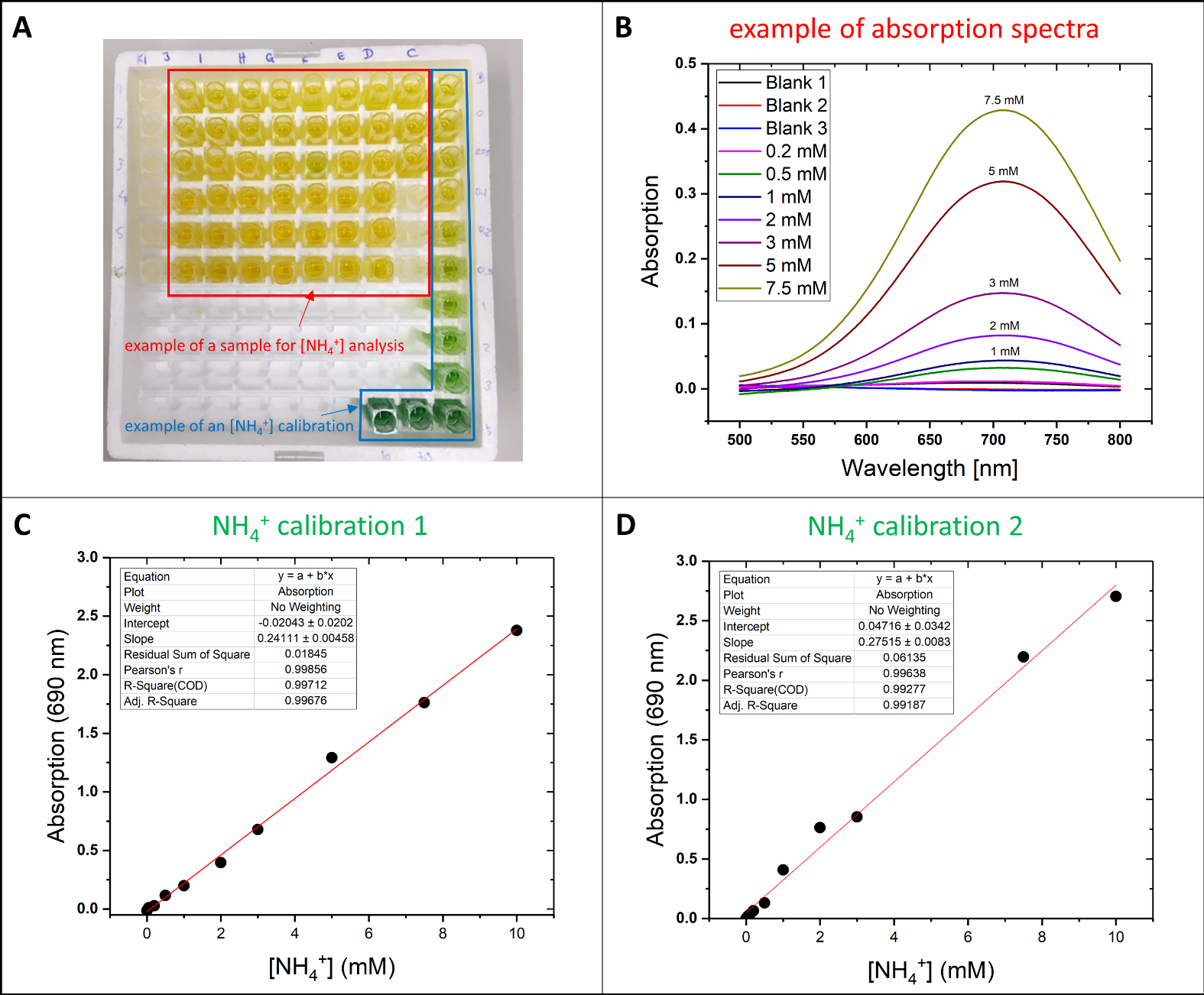
**

**Figure S4.** Example of samples (A) and absorption spectra (B) for calibration and analysis of the ammonium sensitive DET gel, and derived calibration curves (C-D) showing the absorption at 690 nm as a function of the DET gel ammonium concentration (R^2^ ˃ 0.99).

*(h) DGT and DET analysis and interpretation*

The sulphide-binding AgI gels were deployed in the experimental chambers for 8 h during either light exposure of the seagrass leaf canopy or in darkness, and then subjected to CID analysis according to published procedures (Santner et al. 2015; Devries et al. 2003, Teasdale et al. 1999, Robertson et al. 2008, Brodersen et al. 2017). After retrieving the sulphide DGT gels, the protective mesh was removed. The sulphide DGT gels were left untreated, utilizing the contrast of the formed black Ag_2_S of the DGT gel against the pale white background at locations where no sulphide was bound. The sulphide-sensitive gels were then fixed between two transparent PET foils in order to avoid direct contact to the printer, scanned at high resolution of 600 dpi and saved as both colour and greyscale TIFF files. The S_tot_^2-^ concentration and distribution in the experimental sample gels (i.e., biological measurements) were then analysed based on the acquired calibration function (Fig. S3).

The time-averaged S_tot_^2-^ concentration at the sampler-solution interface (i.e., *C_DGT_* concentration of total sulphide) was calculated from the amount of S_tot_^2-^ accumulated during the total gel deployment time (Teasdale et al. 1999; Brodersen et al. 2017). This was done via CID analysis, by quantifying the mass of S_tot_^2-^ taken up by the binding gel as a surface concentration *C_s_* (µg cm^-2^) that can be converted into the concentration at the sampler-exterior solution interface, *C_DGT_*, as:

$$C_{DGT}= C_{s} \frac{\Delta g}{Dt}$$

where *∆g* is the thickness of the diffusion layer overlying the AgI sulphide-binding gel (in this study a nuclepore membrane and then a plankton mesh), *D* is the S_tot_^2-^ diffusion coefficient inside the diffusion layer (13.55 × 10^-6^ cm^2^ s^-1^ at experimental temperature; DGT® Research: www.dgtresearch.com/diffusion-coefficients/) and *t* is the gel deployment time. Note that the sulphide DGT measurement cannot be directly interpreted as an actual S_tot_^2-^ porewater concentration, as the sulphide DGT gel continuously bind S_tot_^2-^ from the exterior solution during deployment and thus the actual porewater concentration at the sampler-sediment interface is decreasing progressively during DGT gel sampling (Santner et al. 2015; Zhang et al. 1995 and 1999; Brodersen et al. 2017).

The DET gels for ammonium determination and analysis were retrieved together with the other gels after 8 hours of exposure. The gels were handled as fast as possible in order to avoid further diffusion within the gel. After retrieval, the gels were cut into 1 x 1 cm squares, and treated according to the calibration procedure described above.

*(i) Experimental Setup*

The experimental chambers consisted of custom-made narrow, transparent glass chambers with a removable front window (Fig. S5). The removable front window enabled easy transplantation of the seagrass in sediment after positioning the DGT/DET gels in the back of the chamber behind a fine, protective plankton mesh (Fig. S5). The experimental chambers with seagrass, sediment and sensors (n = 12) were positioned inside aquaria in an upright position. The glass walls were made of thin glass (~3 mm) to improve the optical properties of the aquaria and the chambers during chemical imaging. Pre-sieved and homogenized sediment was filled into the experimental chambers wherein gently uprooted, selected seagrass plants were transplanted. Natural seawater from the sampling area was used throughout the experiments (temperature of 20°C, salinity of 18).

Light was provided by MIA worldlight lamps (LP300Q-4K.24_RY; MIA Light GmbH, Gronau, Germany) illuminating the seagrass leaf canopy with a scalar photon irradiance (PAR, 400-700 nm) of ~380 µmol photons m^-2^ s^-1^, determined by a scalar irradiance minisensor (US-SQD/L, Walz GmbH, Germany) connected to a calibrated light meter (ULM-500, Walz GmbH, Germany). Water movement and aeration were ensured by means of a submerged water pump and an air-stone connected to an air pump, respectively.

The DGTs and DETs gels for single-analyte determination of total sulphide and ammonium concentrations and distributions were positioned at the back-wall of the experimental chambers, behind a fine mesh (plankton mesh DIN 100-60, mesh size 60 µm, thickness of 50 µm) to enable gel sampling without disturbing the sediment. The single- and dual-analyte planar optodes (i.e., O_2_ and pH imaging) were mounted onto the frontal, removable glass wall.

Seagrass specimens of *Zostera marina* L. were carefully positioned within the sieved natural sediment from the sampling site in the experimental chambers ensuring good contact between the below-ground tissue and the DGT/DET gels, as well as, the optodes during cultivation and measurements. To allow precise positioning and subsequent alignment of the below-ground tissue structures with the colour-coded chemical images, and to facilitate precise sediment sampling for molecular analysis (see below), a grid (1 cm^2^) was made for the front and back glass walls. Additionally, camera pictures including detailed root structure observations and a ruler for guidance were obtained prior to measurements (Fig. S5).

*Z. marina* seagrass and the marine sediment were left undisturbed in the experimental chambers for a minimum of 48 h prior to measurements (i.e., chemical imaging: 48 h (2 days); gel imaging: 96 h (4 days); and sediment sampling: 168 h (7 days)) to ensure establishment of steady state chemical/redox conditions. To facilitate this the sediment and the DGT/DET gels had to be separated by plastic foils (see Fig. S5a), to avoid analyte diffusion into the gels before the sediments biogeochemical processes and chemical conditions had reached steady state conditions (for chemical conditions this is reached after ~5 h; Brodersen et al. 2017). Whereafter, the plastic foils were carefully removed and the gels exposed to sediment and plants. Two sets of DGT/DET gels were deployed, thus enabling steady state sulphide and ammonium gel measurements in both darkness and light without disturbing the sediment during retrieval. To ensure steady state light/dark conditions when measuring, a minimum of 5 h exposure before removal of the plastic foils was allowed. This procedure enabled (*i*) simultaneous steady state measurements of O_2_, pH, total sulphide and ammonium in the seagrass rhizospheres in either light or darkness, as well as (*ii*) determination of high sulphide (i.e., reduced sediment areas), high ammonium (here, within oxidized sediment areas) and seagrass oxidized sediment areas for detailed sediment sampling for molecular analysis of the microbial community composition, as well as the nifH gene abundance and expression (described in detail below). The first series of measurements (n=8) focused on the oxic and high sulphide rhizosphere areas (H2S), the second series of measurements (n=4) focused on the oxidized and high ammonium rhizosphere areas (NH4).

**
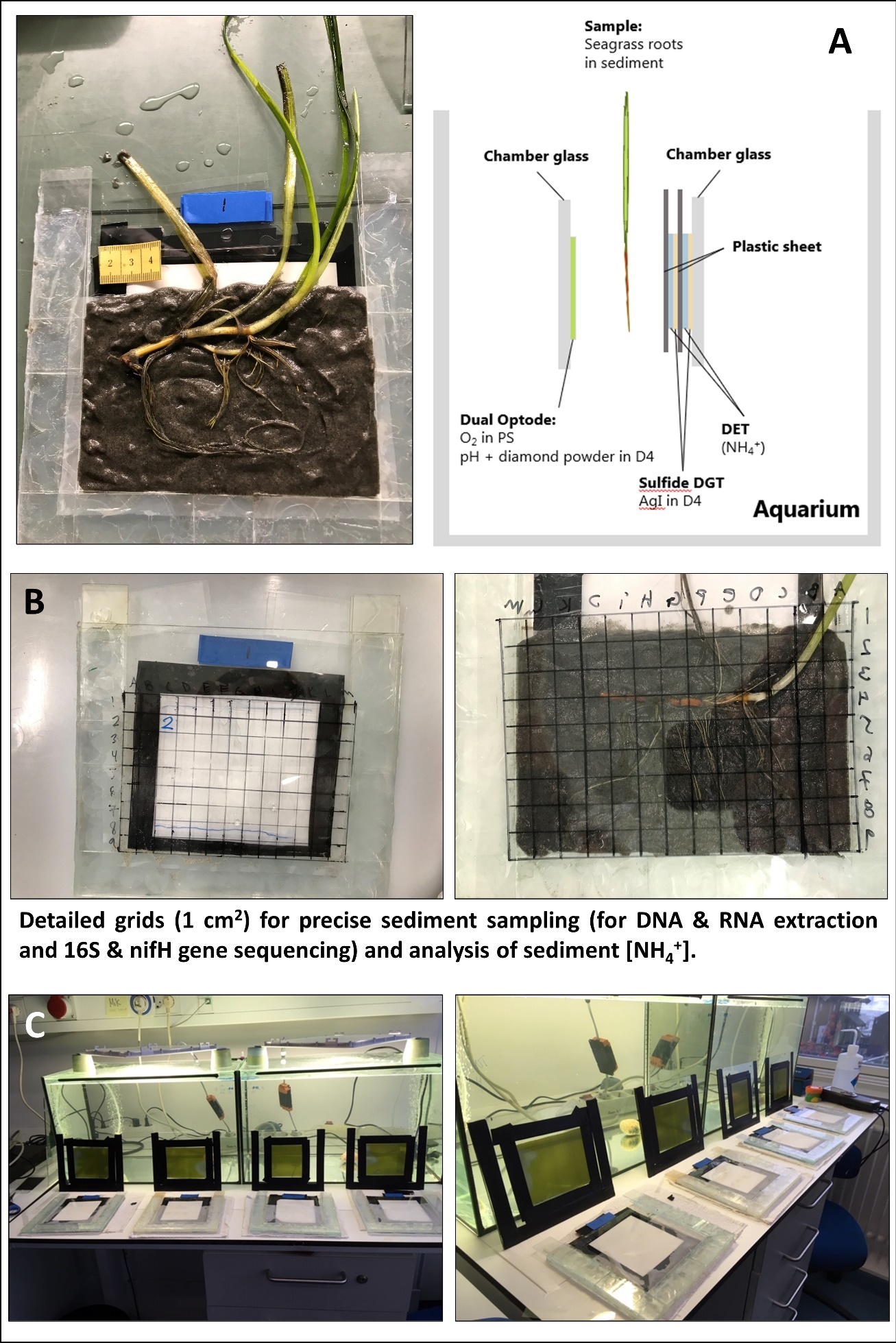
**

**Figure S5.** Experimental setup. Showing the custom-made narrow cultivation chamber (A), the detailed grids for concomitant ammonium analysis and sediment sampling (B), and the narrow chambers with gels and mounted planar optodes (C).

*(j)* *Nucleic acid sampling, extractions and amplicon sequencing*

Sediment was sampled in distinct chemical microenvironments around the roots and rhizome of the seagrasses at the end of the experiments (i.e., bulk sediment, oxidized rhizosphere area, high rhizosphere H_2_S area, and high rhizosphere NH_4_^+^ area) based on the results from the planar optode and DGT/DET measurements, samples were flash frozen in liquid nitrogen and stored at -80°C. This was done using a sterilized spatula and covering foil with grids to carefully avoid cross contamination (Figure S5), enabling precise sediment collection within the 1 cm^2^ grids and at distances of up to only ∼2-3 mm from the below-ground tissue surface when sampling in rhizosphere chemical microenvironments. DNA was extracted from around 0.29 g (0.24-0.33 g) sediment per sample with the Qiagen DNeasy PowerSoil kit according to manufacturer’s instructions. RNA was extracted from around 2 g (0.73-3.11 g) of sediment per samples with the Qiagen RNA Power Soil kit. RNA was stored in multiple aliquots at -80°C. RNA was reverse transcribed with the SuperScript™ IV First-Strand Synthesis System (10 U/µl) and nifH3 reverse primer (0.2 µM final) in 10 µl reactions (Hallstrøm et al. 2022). Controls with RNase-free water instead of the reverse transcriptase were run.

In total three sets of amplicons were generated for subsequent sequencing, 16S DNA, nifH DNA and nifH cDNA. DNA amplicons for the V4/5 region of the 16S rRNA gene were generated with primer pair 515F-Y/926R (Parada et al. 2016). The amplicons for the nifH gene were generated from DNA and cDNA with a nested PCR approach. The primer pair nifH3/nifH4 (Zani et al. 2000) was used in the outer PCR and the primers nifH1/nifH2 (Zehr and McReynolds 1989) with Illumina adapters were applied in the inner PCR.

Around 5 ng of DNA was used in the PCR reactions with the KAPA HiFi Hot Start Ready Mix (Roche, KK2602) and 0.2 µM of the respective primer pair for 16S and outer nifH DNA PCRs. For the cDNA half of the cDNA reaction volume (5 µl) was used as template for the outer nifH PCR reaction. For the inner nifH PCRs 1-2 µl were used from the outer PCRs were used as template (Zehr and Turner, 2001).

Reactions were checked on 1% Agarose gel for correct amplicon size and unspecific amplification. PCR reactions were carried out in triplicate reactions to reduce PCR bias, then pooled and cleaned up using the Geneclean Turbo kit (﻿MP Biomedicals, Germany). Some nifH PCRs with cDNA resulted in unspecific amplification, thus amplicons of the correct size were cut from the 1% agarose gel (molecular grade) and purified with the Invitrogen™ PureLink™ Quick Gel Extraction Kit. All amplicons were indexed in a 10 cycle PCR reaction and purified with the Agencourt AMPure XP beads (Beckman Coulter). DNA concentrations of cleaned amplicons were checked with a PicoGreen assay before pooling samples in equimolar ratios. Samples were sequenced with Illumina MiSeq reagent kit v3 2x300 at the GeoGenetics Sequencing Core, Copenhagen, Denmark. Sequences are deposited in Sequence Read Archive (SRA) under project accession number PRJNA988287.

*(k)* *Sequence processing and taxonomic annotation*

Raw demultiplexed paired-end sequences were processed into amplicon sequence variants (ASVs) using DADA2 (Callahan et al. 2016) implemented in R-4.0.3.

For *nifH* analysis the parameters for ‘filterandTrim’ were: truncLen = c(220,180), maxN = 0, maxEE = c(2,5), truncQ = 2, m.phix = TRUE, trimLeft = 17). Primer sequences were removed by trimLeft, i.e., first 17 bps of the forward and reverse reads. Denoised reads were merged and chimeric sequence mergers removed using the functions “mergePairs” and “removeBimeraDenovo”. Only sequences with a length of 325 to 330 bp were kept. The generated ASVs were translated to amino acid sequences using FrameBot (Wang et al. 2013) and filtered for homologous genes using the NifMAP pipeline (Angel et al. 2018). No homologous genes were observed. Taxonomic ranks were assigned with DIAMOND blastp (Buchfink et al. 2014) using a FrameBot translated *nifH* database (Moynihan 2020) based on the ARB database from the Zehr Lab (version June 2017;<https://www.jzehrlab.com/nifh>). Relationships to the canonical *nifH* clusters (Zehr et al. 2003) were assigned according to Frank et al. (2016). Three nifH RNA samples that had less than 400 reads were removed from the final dataset. Not for all samples nifH cDNA amplicons could be amplified, we assume this is in part due to the low input RNA extractions and inhibitory compounds in the extracts. Read numbers ranged from 407 to 65993 reads (median 2500) for the nifH RNA dataset and from 34439 to 163546 (median 93470).

For 16S analysis the parameters for ‘filterandTrim’ were: truncLen = c(260,210), maxN = 0, maxEE = c(2,5), truncQ = 2, m.phix = TRUE, trimLeft = 20). Primer sequences were removed by trimLeft, i.e., first 20 bps of the forward and reverse reads. Again, denoised reads were merged and chimeric sequence mergers removed using the functions “mergePairs” and “removeBimeraDenovo”. Only sequences with a length of 369 to 375 bp were kept. Read annotation was achieved with the function ‘assignTaxonomy’ using the SILVA database (Silva version 138.1 – UPDATED Mar 10, 2021).

*(l) Molecular data analysis*

Further analyses were performed in R (R Core Team 2022, 4.1.3) and the R package phyloseq (v. 1.38.0; McMurdie and Holmes, 2013) was used for handling sequence abundance tables. Chloroplast and mitochondria sequences were removed from the 16S dataset. Reads numbers for the 16S dataset ranged from 10004 to 52096 (median 33365). Sequence abundance tables were normalized to median sequencing depth. To estimate alpha diversity the Shannon index was calculated with the *estimate_richness* function of phyloseq and the Wilkox test was used to test for statistical differences.

Singletons were excluded from further analysis. The differential abundance analysis was carried out in DESeq2 (Love et al. 2014) on non-standardized reads data by comparing areas of chemical microenvironments (H2S, NH4, oxic) to the bulk sediment (BS). ASVs with less than 50 reads in total were additionally filtered out in each dataset. First the size factors were estimated by applying ‘poscounts’ as it accounts for ASVs missing in some samples and the setting ‘local’ was used for the dispersion estimate. The Wald test was applied to test for significance and the adjusted p-value of < 0.001 was used. Upset plots were produced with UpSetR (1.4.0, Conway et al. 2017), other plots were generated using ‘ggplot2’ (v. 3.3.3; Wickham, 2016: <https://rpkgs.datanovia.com/ggpubr/index.html>) and ggpubr (v. 0.4.0, ﻿Kassambara, A., 2020: <https://github.com/kassambara/ggpubr>).

**SUPPLEMENTARY DATA**

*(m) Oxygen, pH and total sulphide (S_tot_^2-^) concentrations and distribution in the seagrass rhizosphere determined via detailed planar optode imaging and DGT/DET gel measurements – plants 2 and 3.*

**
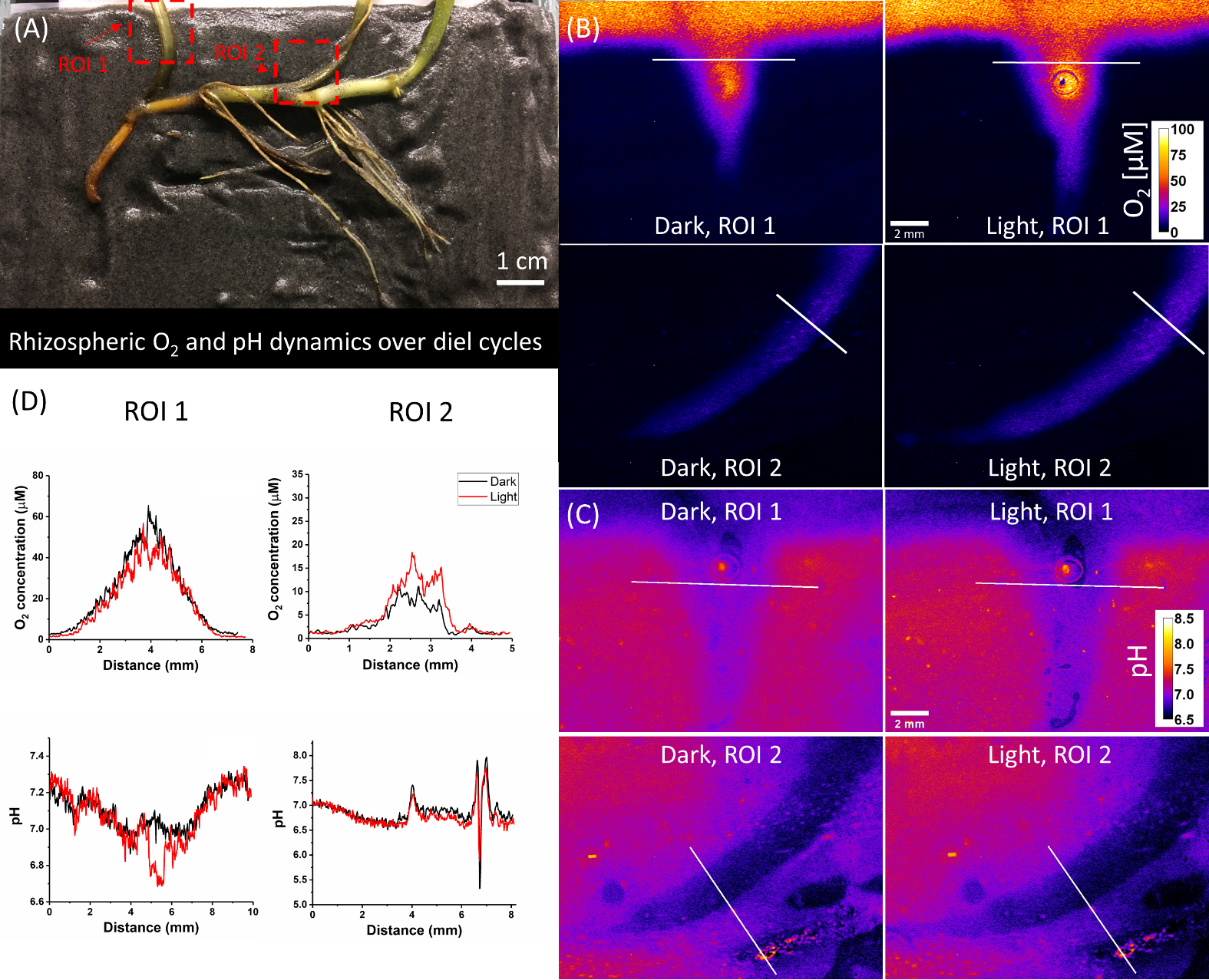
**

**Figure S6.** Oxygen and pH distribution in the seagrass rhizosphere (plant 2). Showing the position of the regions of interest (ROI) in the rhizosphere (A), the rhizospheric O_2_ concentrations around below-ground tissue (B), pH microheterogeneities in the rhizosphere (C), and extracted cross-tissue line profiles of the [O_2_] and pH (D). Extracted line profiles are shown on images in panel B-C as white line. For analysis of the microbial community composition within oxic microenvironments all areas in close proximity to the plant below-ground tissues were sampled.

**
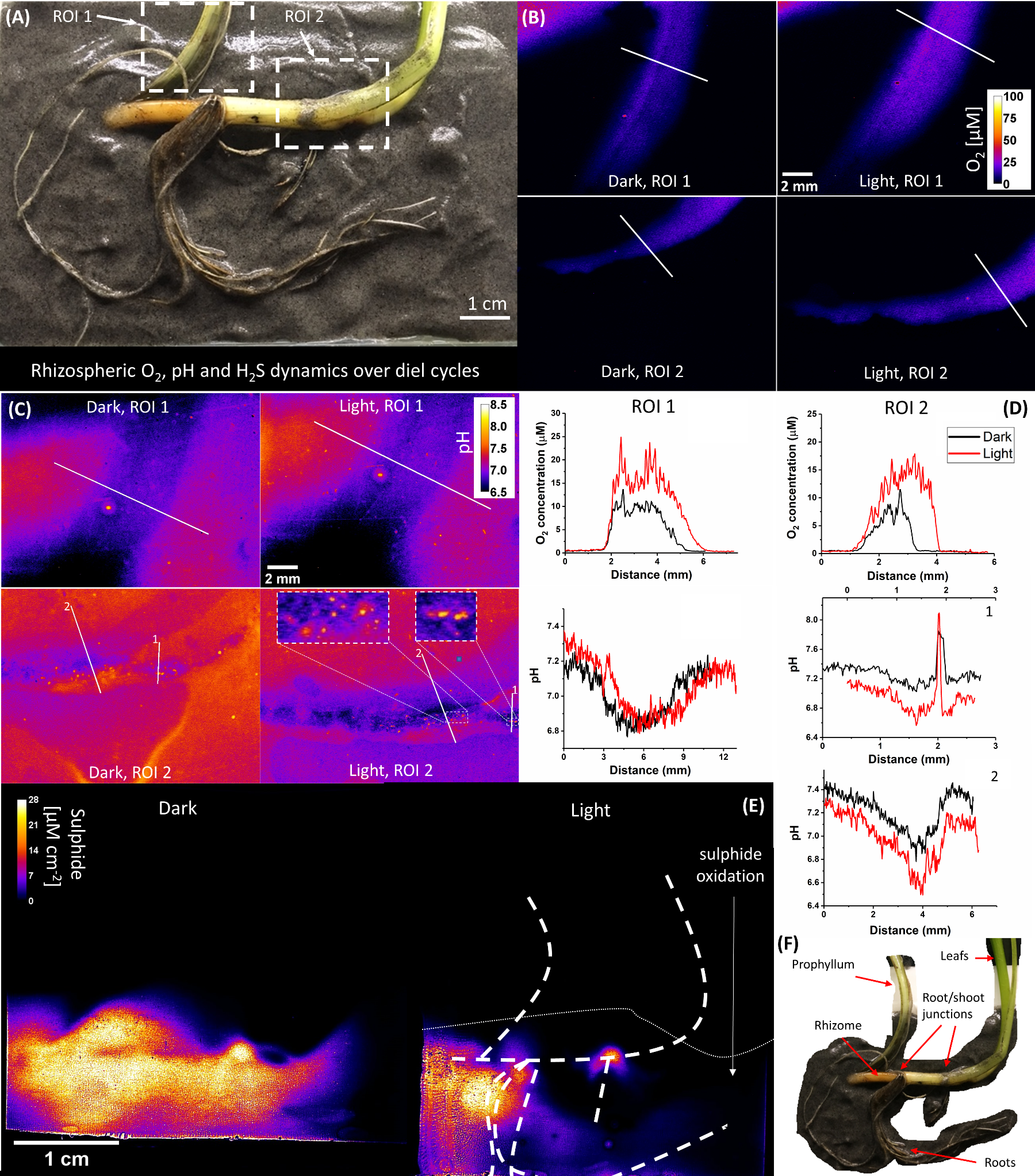
**

**Figure S7.** O_2_, pH and S_tot_^2-^ distribution in the seagrass rhizosphere (plant 3). Showing the position of the regions of interest (ROI) in the rhizosphere (A), the rhizospheric O_2_ concentrations around below-ground tissue (B), the pH microheterogeneities in the rhizosphere with close-up images of high-pH rhizospheric areas (C), extracted cross-tissue line profiles of the dissolved O_2_ concentration and pH distributions (D) (extracted line profiles are showed on images in panel B-C as white line), the total sulphide distribution (c_DGT_) in the seagrass rhizosphere (E) where the dotted white line marks the sediment/water interface in light, and the structural position of the seagrass plant (F) that is also marked in panel E. For analysis of the microbial community composition within oxic and high sulphide microenvironments all areas in close proximity to the plant below-ground tissues were sampled.

*(n) Ammonium concentration and distribution in the seagrass rhizosphere – plants 5-8.*

Figure S8 is shown on the following page 🡪

**
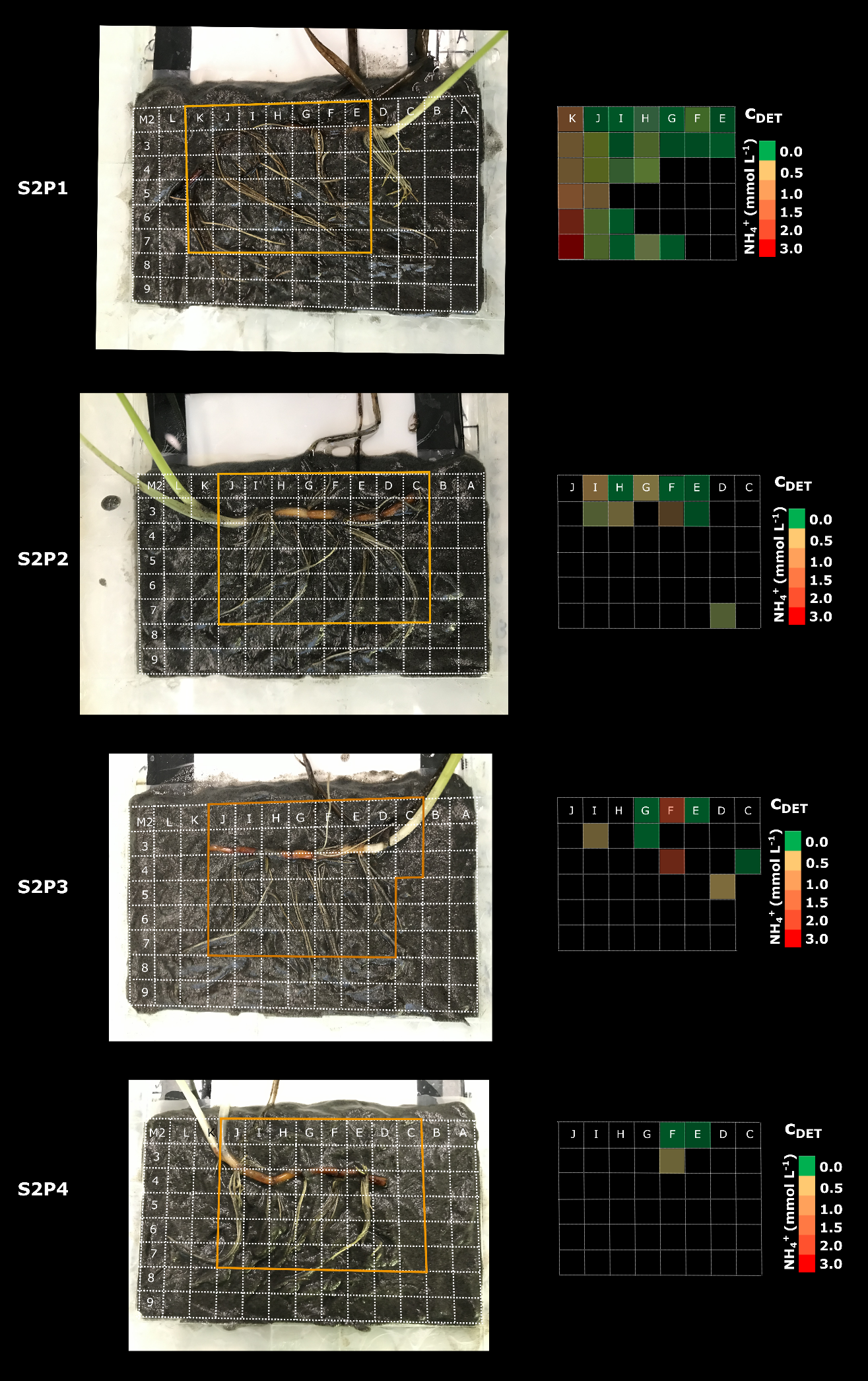
**

**Figure S8.** The c_DET_ ammonium (NH_4_^+^) concentration and distribution in the seagrass rhizosphere (plant replicates 5-8). Images on the left shows the position of the plant structure and the gel. Images on the right shows the ammonium c_DET_ concentration around the below-ground tissue. The legend depicts the ammonium c_DET_ concentration, where green is low and red high concentration. Black colour code is below the detection limit.

*(o) nifH community composition subclusters*

**
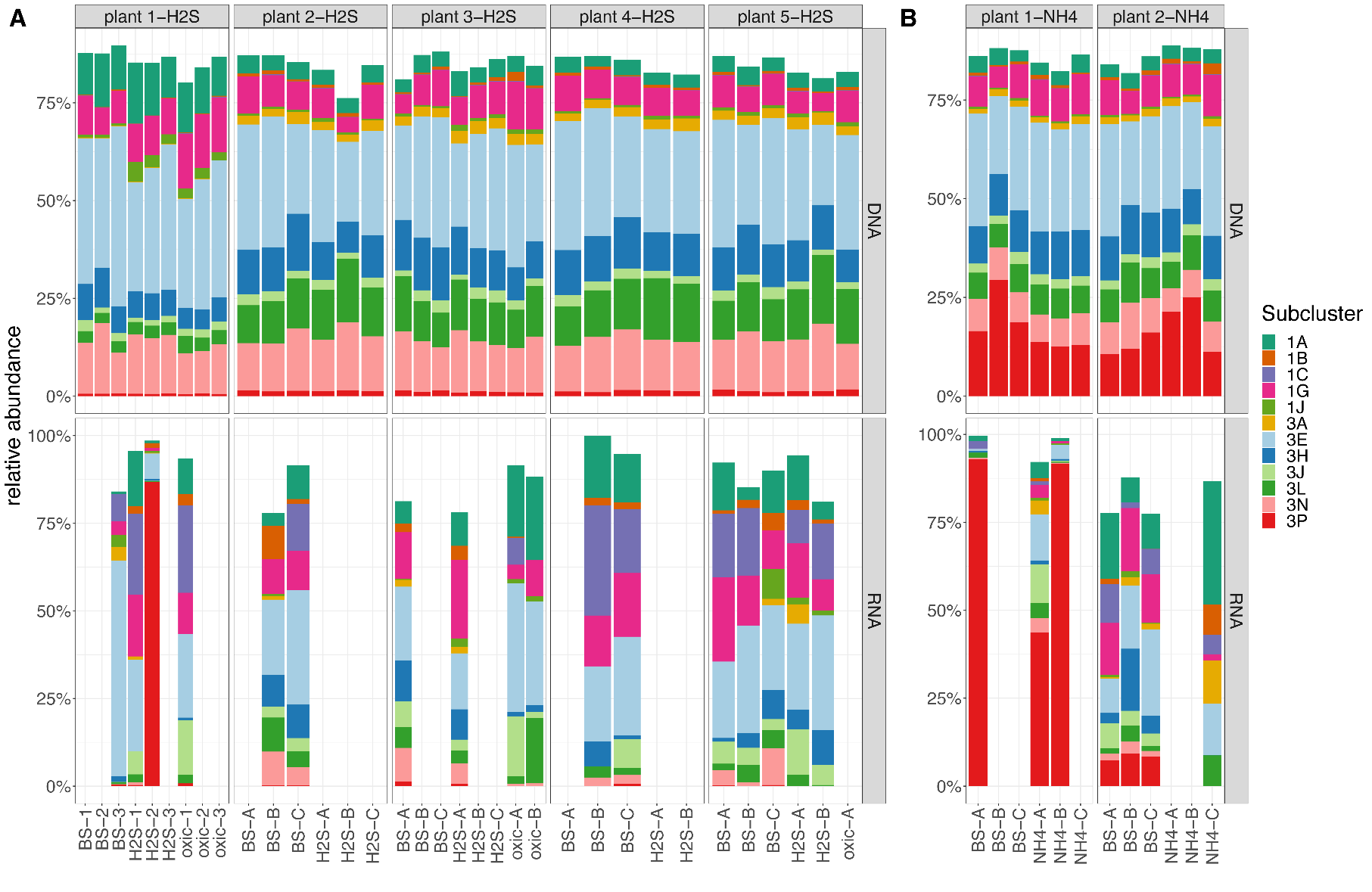
**

**Figure S9.** Relative abundance of nifH DNA (top) and RNA (bottom) amplicon ASVs with the top 12 nifH subcluster affiliation. BS: Bulk Sediment, H2S: rhizosphere area of high H_2_S, oxic: oxidized rhizosphere area, NH4: rhizosphere area of high NH_4_^+^. First experiments focused on the high H_2_S areas around the seagrass roots and rhizome **(A)**. Second set of experiments targeted the high NH_4_^+^ areas of the seagrass rhizosphere **(B)**.

*(p) 16S and nifH unique ASVs*


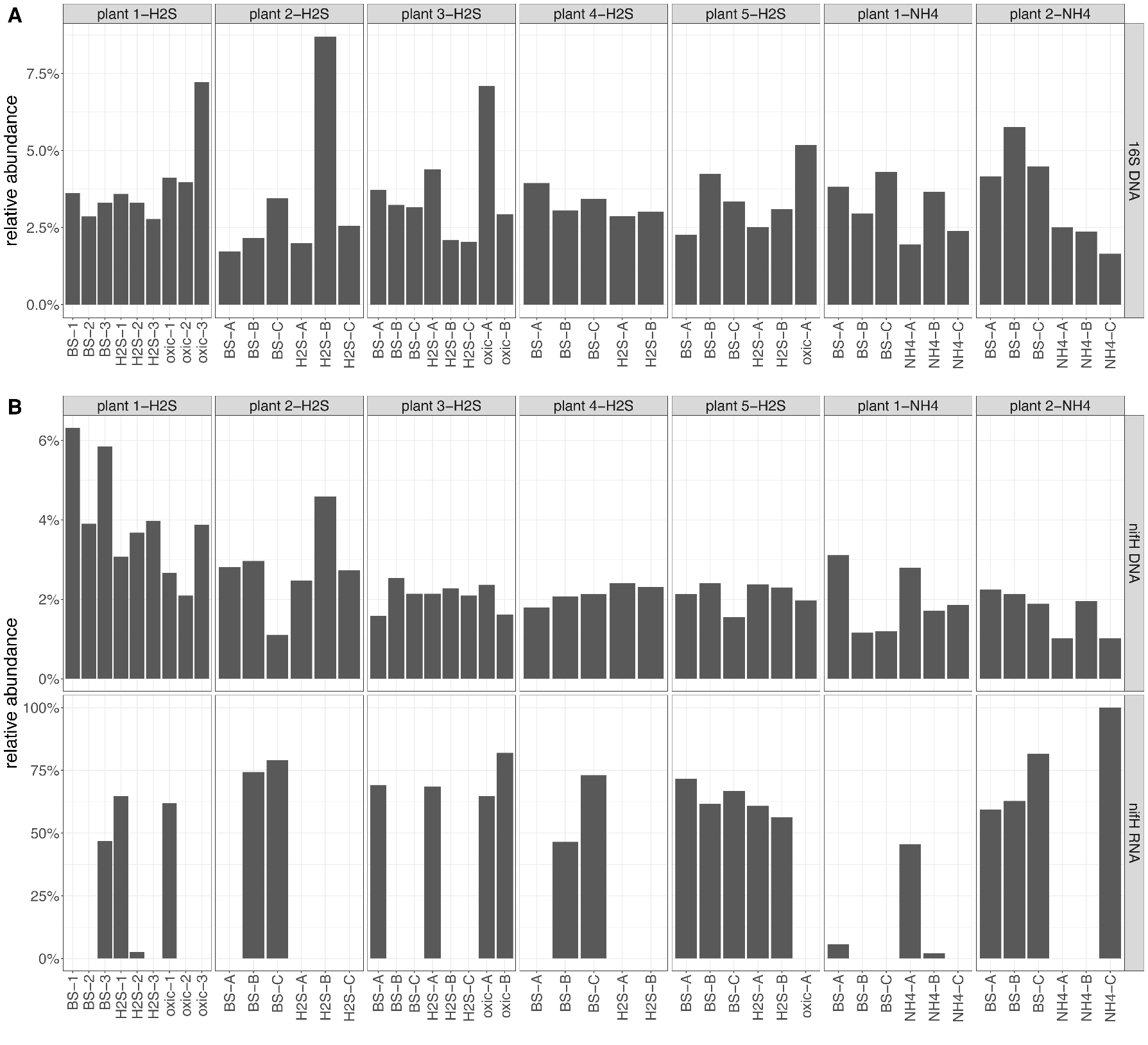


**Figure S10.** Relative abundance of the 16S and nifH DNA and RNA unique ASVs for the selected regions of interest: bulk sediment (BS), oxidized rhizosphere area (oxic), high rhizosphere H_2_S area (H2S) and high rhizosphere NH_4_^+^ area (NH4).

*(q) Shannon diversity index*


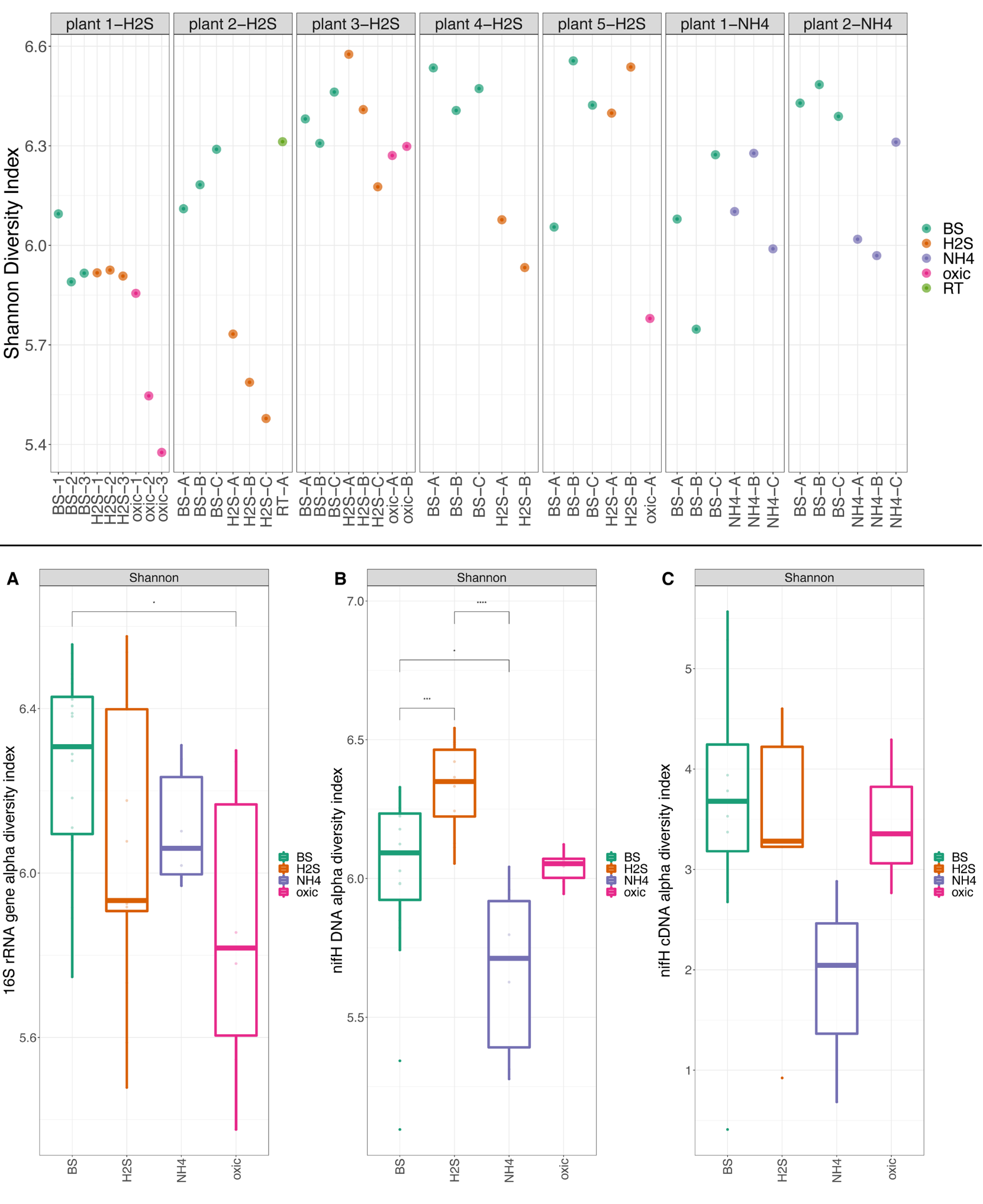


**Figure S11.** Alpha diversity of the 16S rRNA gene estimated by Shannon diversity index across plants and areas (top). Alpha diversity as Shannon diversity index for ASVs across areas and datasets 16S rRNA gene ASVs (A), nifH DNA ASVs (B), and nifH cDNA ASVs (C). Stars indicate p-values (* < 0.05, *** < 0.001, **** < 0.0001) as determined with pairwise Wilcox test (bottom). BS = bulk sediment, H2S = high H_2_S rhizosphere area, NH4 = high ammonium rhizosphere area, and oxic = oxidized rhizosphere area.

**Table S1:** Number of ASVs after each cleaning step.


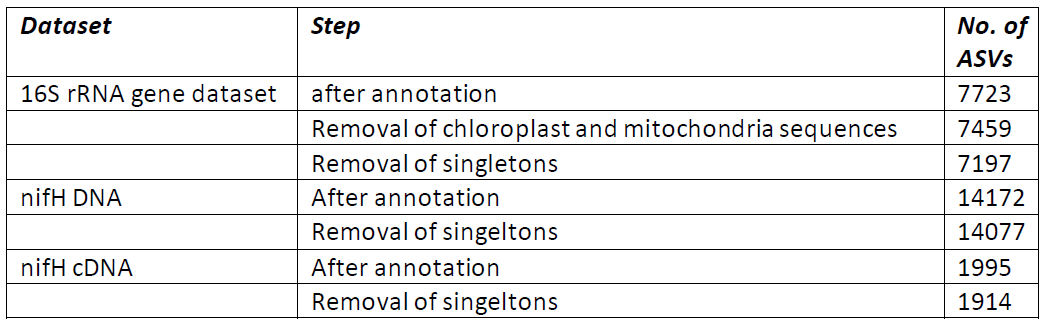


Full taxonomy tables are available at *figshare*. Link: [Supplementary Files: Seagrass-mediated rhizosphere redox gradients are linked with ammonium accumulation driven by diazotrophs (figshare.com)](https://figshare.com/articles/dataset/_b_Supplementary_Files_Seagrass-mediated_rhizosphere_redox_gradients_are_linked_with_ammonium_accumulation_driven_by_diazotrophs_b_/24996404)

**REFERENCES**

Angel R, Nepel M, Panhölzl C, Schmidt H, Herbold CW, Eichorst SA, Woebken D. (2018). Evaluation of Primers Targeting the Diazotroph Functional Gene and Development of NifMAP – A Bioinformatics Pipeline for Analyzing *nifH* Amplicon Data. *Frontiers in Microbiology* 9: 1-15.

Brodersen KE, Koren K, Moßhammer M, Ralph PJ, Kühl M, Santner J. (2017). Seagrass-Mediated Phosphorus and Iron Solubilization in Tropical Sediments. *Environmental Science & Technology* **51** (24):14155-14163.

Buchfink B, Xie C, Huson DH. (2015). Fast and sensitive protein alignment using DIAMOND. *Nature Methods* 12 (1):59-60.

Callahan BJ, McMurdie PJ, Rosen MJ, Han AW, Johnson AJA, Holmes SP. (2016). DADA2: High-resolution sample inference from Illumina amplicon data. *Nature Methods* 13 (7):581-583.

Conway JR, Lex A, Gehlenborg N. (2017). UpSetR: an R package for the visualization of intersecting sets and their properties. *Bioinformatics* 33 (18):2938-2940.

Devries C, Wang F. (2003). In situ two-dimensional high-resolution profiling of sulfide in sediment interstitial waters. *Environmental Science & Technology*, **37:** 792-797.

Frank IE, Turk-Kubo KA, Zehr JP. (2016). Rapid annotation of nifH gene sequences using classification and regression trees facilitates environmental functional gene analysis. *Environmental Microbiology Reports* 8 (5):905-916.

Hallstrøm S, Benavides M, Salamon ER, Evans CW, Potts LJ, Granger J, Tobias CR, Moisander PH, Riemann L. (2022). Pelagic N_2_ fixation dominated by sediment diazotrophic communities in a shallow temperate estuary. *Limnology and Oceanography* 67 (2):364-378

Love MI, Huber W, Anders S. (2014). Moderated estimation of fold change and dispersion for RNA-seq data with DESeq2. *Genome Biology* 15 (12):550.

McMurdie PJ, Holmes S. (2013). phyloseq: An R Package for Reproducible Interactive Analysis and Graphics of Microbiome Census Data. *PLOS ONE* 8 (4):e61217.

Moßhammer M, Strobl M, Kühl M, Klimant I, Borisov SM, Koren K. (2016). Design and application of an optical sensor for simultaneous imaging of pH and dissolved O_2_ with low cross-talk. *ACS Sensors* **1** (6):681-687.

Moynihan, M. A. (2020). NifHdada2 GitHub Repository*.* (version v. 1.1.0). Zenodo. <http://doi.org/10.5281/zenodo.3958370>.

Parada AE, Needham DM, Fuhrman JA (2016) Every base matters: assessing small subunit rRNA primers for marine microbiomes with mock communities, time series and global field samples. Environmental microbiology 18 (5):1403-1414

R Core Team (2022). R: A language and environment for statistical computing. R Foundation for Statistical Computing, Vienna, Austria. https://www.R-project.org/.

Robertson D, Teasdale PR, Welsh DT. (2008) A novel gel-based technique for the high resolution, two-dimensional determination of iron (II) and sulfide in sediment. *Limnology and Oceanography : Methods,* **6:** 502-512.

Santner J, Larsen M, Kreuzeder A, Glud RN. (2015). Two decades of chemical imaging of solutes in sediments and soils - a review. *Analytica Chimica Acta,* **878:** 9-42.

Teasdale PR, Hayward S, Davison W. (1999). In situ, High-Resolution Measurement of Dissolved Sulfide Using Diffusive Gradients in Thin Films with Computer-Imaging Densitometry. *Analytical Chemistry,* **71:** 2186-2191.

Wang Q, Quensen JF, Fish JA, Lee TK, Sun Y, Tiedje JM, Cole JR. (2013). Ecological Patterns of *nifH* Genes in Four Terrestrial Climatic Zones Explored with Targeted Metagenomics Using FrameBot, a New Informatics Tool. *mBio* 4 (5):e00592-00513.

Wickham, H. (2016). *Ggplot2: Elegant Graphics for Data Analysis*. Springer-Verlag New York. [https://ggplot2.tidyverse.org](https://ggplot2.tidyverse.org/).

Zani S, Mellon MT, Collier JL, Zehr JP. (2000). Expression of nifH genes in natural microbial assemblages in Lake George, New York, detected by reverse transcriptase PCR. Applied and *Environmental Microbiology* 66 (7):3119-3124

Zehr JP, Jenkins BD, Short SM, Steward GF. (2003). Nitrogenase gene diversity and microbial community structure: a cross-system comparison. *Environmental Microbiology* 5 (7):539-554.

Zehr JP, McReynolds LA (1989) Use of degenerate oligonucleotides for amplification of the nifH gene from the marine cyanobacterium Trichodesmium thiebautii. *Applied and Environmental Microbiology* 55 (10):2522-2526.

Zehr JP, Turner PJ (2001) Nitrogen fixation: Nitrogenase genes and gene expression. In: Methods in Microbiology, vol 30. Academic Press, pp 271-286.

Zhang H, Davison W. (1995). Performance characteristics of diffusion gradients in thin films for the in situ measurement of trace metals in aqueous solution, *Analytical Chemistry,* **67** 3391–3400.

Zhang H, Davison W. (1999). Diffusional characteristics of hydrogels used in DGT and DET techniques. *Analytica Chimica Acta*, **398**: 329–340.
